# New species of the genus *Mesocletodes* Sars, 1909 from the deep Gulf of California (Copepoda, Harpacticoida)

**DOI:** 10.3897/zookeys.751.20387

**Published:** 2018-04-19

**Authors:** Samuel Gómez

**Affiliations:** 1 Instituto de Ciencias del Mar y Limnología, Unidad Académica Mazatlán, Universidad Nacional Autónoma de México; Joel Montes Camarena s/n, Fracc. Playa Sur, Mazatlán, 82040, Sinaloa, México

**Keywords:** Argestidae, *Mesocletodes*, deep sea, new species, taxonomy

## Abstract

Investigations on the effects of the oxygen minimum zone on the distribution, abundance, and diversity of deep-sea benthic and pelagic fauna of the Gulf of California and Eastern Tropical Pacific has received attention recently. However, very little is known about the diversity of deep-sea benthic harpacticoids from this region, and only three species, *Ancorabolus hendrickxi* Gómez & Conroy-Dalton, 2002, *Ceratonotus elongatus* Gómez & Díaz, 2017 and *Dendropsyllus californiensis* Gómez & Díaz, 2017, have been described so far. The genus *Mesocletodes* Sars, 1909 is one of the most common and abundant genera of deep-sea harpacticoids. This genus has been traditionally subdivided into two species groups, the *abyssicola* and the *inermis* groups, based on the presence/absence of a dorsal cuticular process on the cephalothorax and anal somite, but some species have been reported to deviate from this scheme. As a result of their investigations, other researchers proposed the monophyly of the *abyssicola* group, and suggested the probable monophyly of two other species-groups. In this paper, the descriptions of three new species of the genus *Mesocletodes* from the deep sea of the Gulf of California are presented with some notes on their relationships with other species. Some comments on the monophyly of the genus are given.

## Introduction

Great effort has been deployed since the late 1980’s to study the biodiversity of the deep sea of the Gulf of California and Eastern Tropical Pacific (a complete list of contributions is available upon request). Extensive oceanographic campaigns (Talud I-XIII cruises) on board the research vessel “El Puma” of the Universidad Nacional Autónoma de México (UNAM) have been carried out from 1989 to 2009 in the frame of several research projects to better understand the effects of the oxygen minimum zone on the distribution, abundance and diversity of deep-sea benthic and pelagic fauna (crustaceans, molluscs, echinoderms, fishes and polychaetes among others) of the Gulf of California and Eastern Tropical Pacific (Hendrickx 2012). One of the components studied during Talud IV- Talud XIII cruises was the meiofauna, with especial attention to benthic harpacticoid copepods. Preliminary personal observations revealed a high diversity of harpacticoids. However, only three species of the family Ancorabolidae, *Ancorabolus hendrickxi* Gómez & Conroy-Dalton, 2002, *Ceratonotus elongatus* Gómez & Díaz, 2017 and *Dendropsyllus californiensis* Gómez & Díaz, 2017 have been described so far from the deep sea of the Gulf of California.

The genus *Mesocletodes* Sars, 1909 is one of the most common and abundant genera of the family Argestidae in deep-sea samples (Menzel 2011). Traditionally, two species-groups are recognized within the genus, and Bodin (1968, 1997) proposed the subdivision of the genus *Mesocletodes* into the *abyssicola* group and the *inermis* group, based on the presence/absence of a simple dorsal cuticular process on the cephalothorax and anal somite, but pointed out that that such subdivision has no taxonomic value. However, with the discovery of some new species, it was found that some deviate from Bodin’s (1968, 1997) scheme. For example, Por (1986: 95) suggested that *M. opoteros* Por, 1986, described without a dorsal process on the cephalothorax but with a dorsal process on the anal somite, could well belong to a different species-group within the genus, and suggested that Bodin’s (1968, 1997) division of this genus into species-groups could change with the discovery of new species. A similar condition has been observed for *M. fladensis* Wells, 1965 and *M. angolaensis* Menzel & George, 2009. However, Menzel and George (2009) did not follow Bodin’s (1968, 1997) and Por’s (1986) views. Instead, they suggested the monophyly of the *abyssicola* group based on the elongation of the caudal rami, and on the presence of a dorsal process on the cephalothorax and on the anal somite, and pooled all the species of *Mesocletodes*, with a dorsal cuticular process either on the cephalothorax or on the anal somite, or both, and with long or short caudal rami, in the *abyssicola* group, arguing that the deviation of Bodin’s (1968, 1997) scheme (presence of a dorsal process on the cephalothorax or on the anal somite only), and the shorter caudal rami observed in some species could eventually be regarded as secondary reductions (Menzel and George 2009: 253). Additionally, Menzel and George (2009) suggested the monophyly of, at least, two other groups of species, viz. those in which the males lack mouth parts (Menzel and George 2009: 253–254), and those with bifid dorsal processes on the P3-P5-bearing somites, and on the posterior half of the female double genital-somite (Menzel and George 2009: 254).

In this paper three new species of *Mesocletodes* from the deep sea of the Gulf of California are proposed. Additionally, some comments on the monophyly of *Mesocletodes* are provided.

## Materials and methods

Sediment samples for meiofaunal analyses were taken in August 2000 in the Southern Gulf of California from Carmen basin to off Nayarit State, and in February 2007 in the Southern Trough of Guaymas Basin, during Talud IV and Talud X cruises, respectively, on board the research vessel “El Puma” of the Universidad Nacional Autónoma de México (UNAM). Sediment samples were collected at depths ranging from about 520 m to 2120 m during Talud IV cruise using a multiple sediment corer equipped with six cores of 30 cm in length and sampling surface of 3.9 cm^2^, and from about 379 m to 1902 m during Talud X cruise using a box corer from which triplicate sub-samples were taken with 69 cm^2^ cores of 20 cm in length. The upper 3 cm layer of sediment was preserved in 70% alcohol, sieved through 500 and 38 µm sieves to separate macro- and meiofauna, and stained with Rose Bengal. Meiofauna was sorted at a magnification of 40× using an Olympus SZX12 stereomicroscope equipped with DF PLAPO 1× objective and WHS10× eyepieces, and harpacticoid copepods were stored separately in 1 ml vials with 70% ethanol. Illustrations and figures were made from whole individuals and its dissected parts using a Leica DMLB microscope equipped with L PLAN 10× eyepieces, N PLAN 100× oil immersion objective, and drawing tube. The dissected parts were mounted on separate slides using lactophenol as mounting medium. Huys and Boxshall (1991) and Menzel and George (2009) were followed for general terminology. Huys (1996) was followed for the subapical tubulate extensions (STE).

Abbreviations used in the text:

**acro** acrotheke;

**ae** aesthetasc;

**ENP** endopod;

**EXP** exopod;

**EXP (ENP)1 (2,3)** first (second, third) exopodal (endopodal) segment;

**P1–P6** first to sixth legs;

**se** pinnate, naked, setiform;

**sp** spinose, spiniform;

**STE** subapical tubulate extension.

The type material was deposited in the Copepoda collection of the Instituto de Ciencias del Mar y Limnología, Unidad Académica Mazatlán (**ICML-EMUCOP**). The map showing the sampling locations where the new species were found were prepared with GeoMapApp (http://www.geomapapp.org/) and the Global Multi-Resolution Topography (GMRT) default basemap (Ryan et al. 2009).

## Taxonomy

### Family Argestidae Por, 1986a

#### Genus *Mesocletodes* Sars, 1909

##### Mesocletodes brevisetosus

Taxon classificationAnimaliaHarpacticoidaArgestidae

sp. n.

http://zoobank.org/8E54143A-D029-4D06-BC1E-A5EBFFF9847E

###### Material examined.

One female holotype as follows: habitus, left antennule and right antenna left intact and preserved in alcohol (ICML-EMUCOP-270800-02), right antennule, left antenna, mandibles, maxillules, maxillae and maxillipeds dissected and mounted onto four slides (ICML-EMUCOP-270800-01); Talud IV cruise; August 27, 2000; coll. S. Gómez.

###### Type locality.

**Figure 1.**
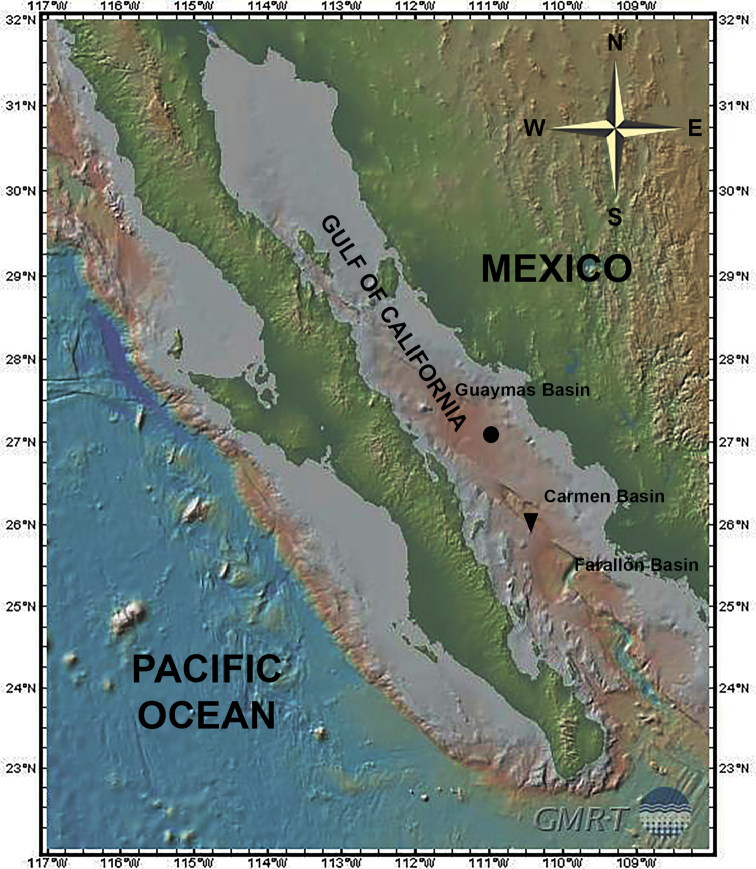
Sampling sites and type localities of *Mesocletodes simplex* sp. n. (full circle), and *M. brevisetosus* sp. n. and *M. unisetosus* sp. n. (full triangle). Figure prepared with GeoMapApp (http://www.geomapapp.org/) and the Global Multi-Resolution Topography (GMRT) default basemap (Ryan et al. 2009).

Southern Carmen Basin, Gulf of California, México (25°54.7'N, 110°11'W), 2018 m depth (see Fig. 1); coll. S. Gómez.

###### Diagnosis (based on female only).

Body subcylindrical. Cuticula of body surface reticulated. Free thoracic somites and urosomites, except for anal somite, with posterior margin coarsely serrated. Cephalothorax with dorsal process curved posteriorly. P3–P5-bearing somites and second half of genital-double somite with bifid cuticular processes dorsally. Genital somite and third urosomite incompletely fused dorsolaterally. Anal somite with upward bifid cuticular process dorsally. Caudal rami 17 times as long as wide in lateral view, with seven setae. Antennule octa-segmented; second segment with strong protrusion bearing one strong element. Antenna with basis, with uni-segmented exopod bearing two setae subequal in length. Gnathobase of mandible with grinding face, palp uni-segmented, endopodal lobe with four setae. P2–P4 ENP1 with inner seta. P1–P4 ENP2 with 3, 3, 2, 2, setae respectively. P5 endopodal lobe represented by two setae; inner seta of the P5 EXP very small, issuing subapically.

###### Description of female.

**Figure 2.**
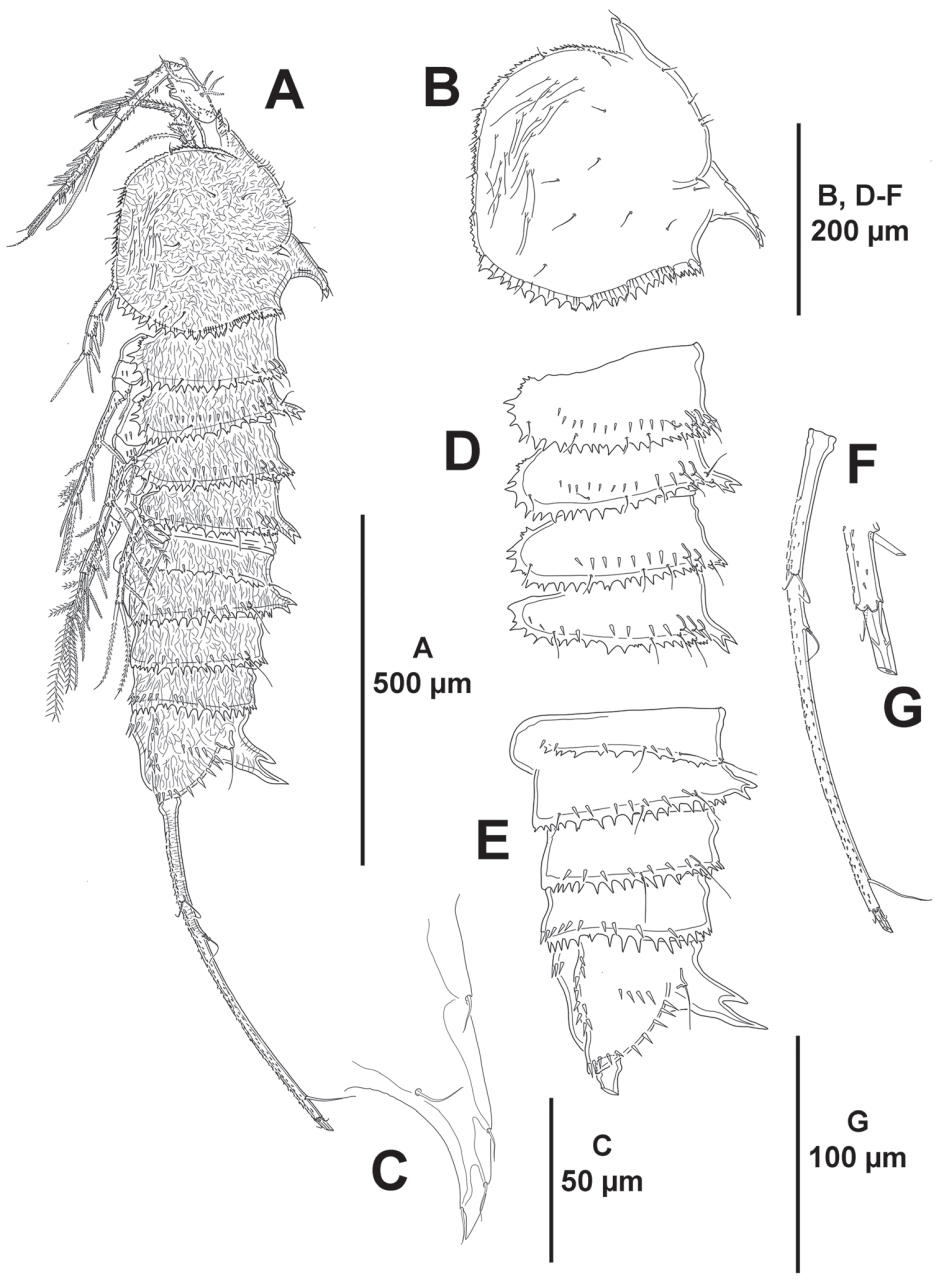
*Mesocletodes brevisetosus* sp. n., female holotype. **A** habitus, lateral **B** cephalothorax, lateral, reticulated pattern omitted **C** dorsal cuticular process of cephalothorax showing situation of sensilla, reticulated pattern omitted **D** P2–P5-bearing somites, lateral, reticulated pattern omitted **E** genital double-somite, fourth and fifth urosomites, and anal somite, lateral, reticulated pattern omitted **F** left caudal ramus, lateral, reticulated pattern omitted **G** distal part of left caudal ramus, lateral, reticulated pattern omitted.

**Figure 3.**
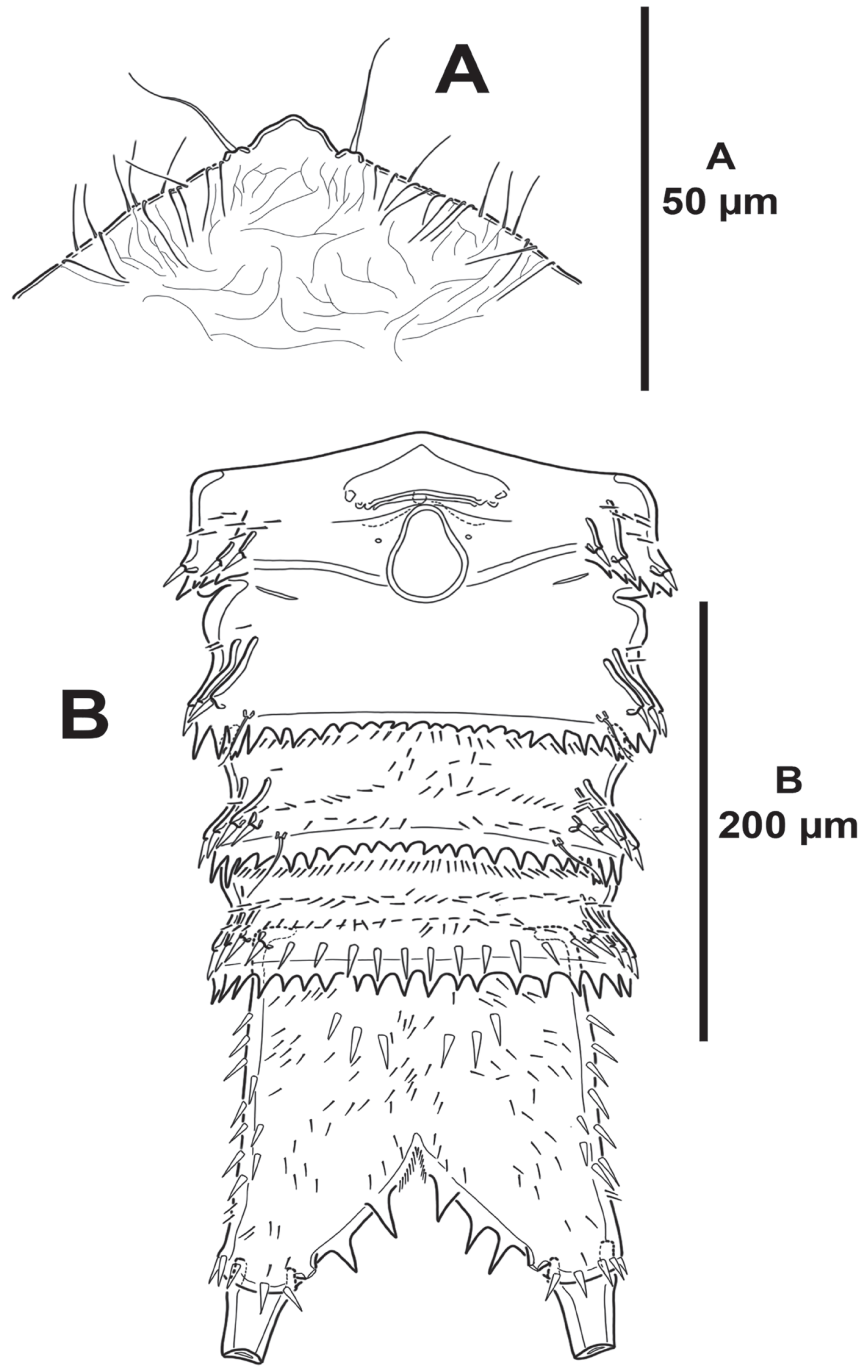
*Mesocletodes brevisetosus* sp. n., female holotype. **A** rostrum, dorsal **B** urosome, ventral, P5-bearing somite omitted.

*Body*: total length 1420 µm measured from anterior margin of rostrum to posterior margin of caudal rami, subcylindrical, tapering slightly posteriorly, without clear demarcation between prosome and urosome, cuticula of body surface reticulated (Fig. 2A); P2–P5-bearing somites (Fig. 2D), both halves of genital double-somite and fourth and fifth urosomites (Fig. 2E) with posterior margin coarsely serrated; lateral margin of cephalothorax less coarsely serrated (Fig. 2B). Rostrum (Figs 2A–B, 3A) fused to cephalothorax, with two sensilla. Cephalothorax (Fig. 2A–B) 0.2 times as long as entire body length; with small sensilla as shown, and with exceedingly long lateral sensilla (Fig. 2B); with dorsal process curved posteriorly (Fig. 2A, B), the latter with sensilla as shown (Fig. 2C). Body somites with posterior transverse longitudinal row of spinules (Fig. 2A, D, E). P2-bearing somite without, P3–P5-bearing somites with bifid cuticular processes dorsally, of P4-bearing somite smallest, of P3- and P5-bearing somites subequal (Fig. 2D). Genital somite and third urosomite (genital double-somite) incompletely fused dorsolaterally (Fig. 2E), posterior margin of genital somite indicated by suture with few spinules and sensilla, and serrated posterior margin, completely fused ventrally (Fig. 3B); first half of genital double-somite without cuticular process dorsally (Fig. 2A, E), with few lateral spinules close to posterior margin (Fig. 2E), ventrally with medial genital field (Fig. 3B) and with some spinules close to lateral margins, second half with bifid cuticular process dorsally (Fig. 2A, E), with lateral spinules along posterior margin, serrated posterior margin between pair of ventral sensilla less pronounced (Figs 2E, 3B). Dorsal and lateral surface of fourth urosomite with short row of small spinules, with few sensilla, posterior margin serrated (Fig. 2A, E), ventrally with posterior serrated margin slightly less pronounced between pair of ventral sensilla, with minute spinules as shown (Fig. 3B). Fifth urosomite (Fig. 2E) with less spinules than in preceding somite, without sensilla, posterior margin equally serrated along entire margin, ventrally (Fig. 3B) with continuous spinular row close to posterior margin, with minute spinules as shown.

*Anal somite* (Figs 2A, E, 3B) quadrate, as long as two preceding somites combined; with lateral (Fig. 2A, E) and ventral (Fig. 3B) spinules, with upward bifid cuticular process dorsally, flanked by pair of sensilla, posterior tip of cuticular process longer than anterior (Fig. 2A, E).

*Caudal rami* slender, exceedingly elongated, 17 times as long as wide in lateral view, as long as P4-bearing somite and entire urosome combined, gently curved upwards from lateral view, covered with small spinules (Fig. 2A, F); with seven elements as follows: seta I and II in distal part of first third of ramus, the former ventral to and smaller than the latter (Fig. 2F); seta III situated subdistally on dorsal surface (Fig. 2G); seta IV and VI small, subequal in length, arising at outer and inner distal corners, respectively; seta V longest; dorsal seta VII tri-articulated, situated on anterior part of second third of ramus.

**Figure 4.**
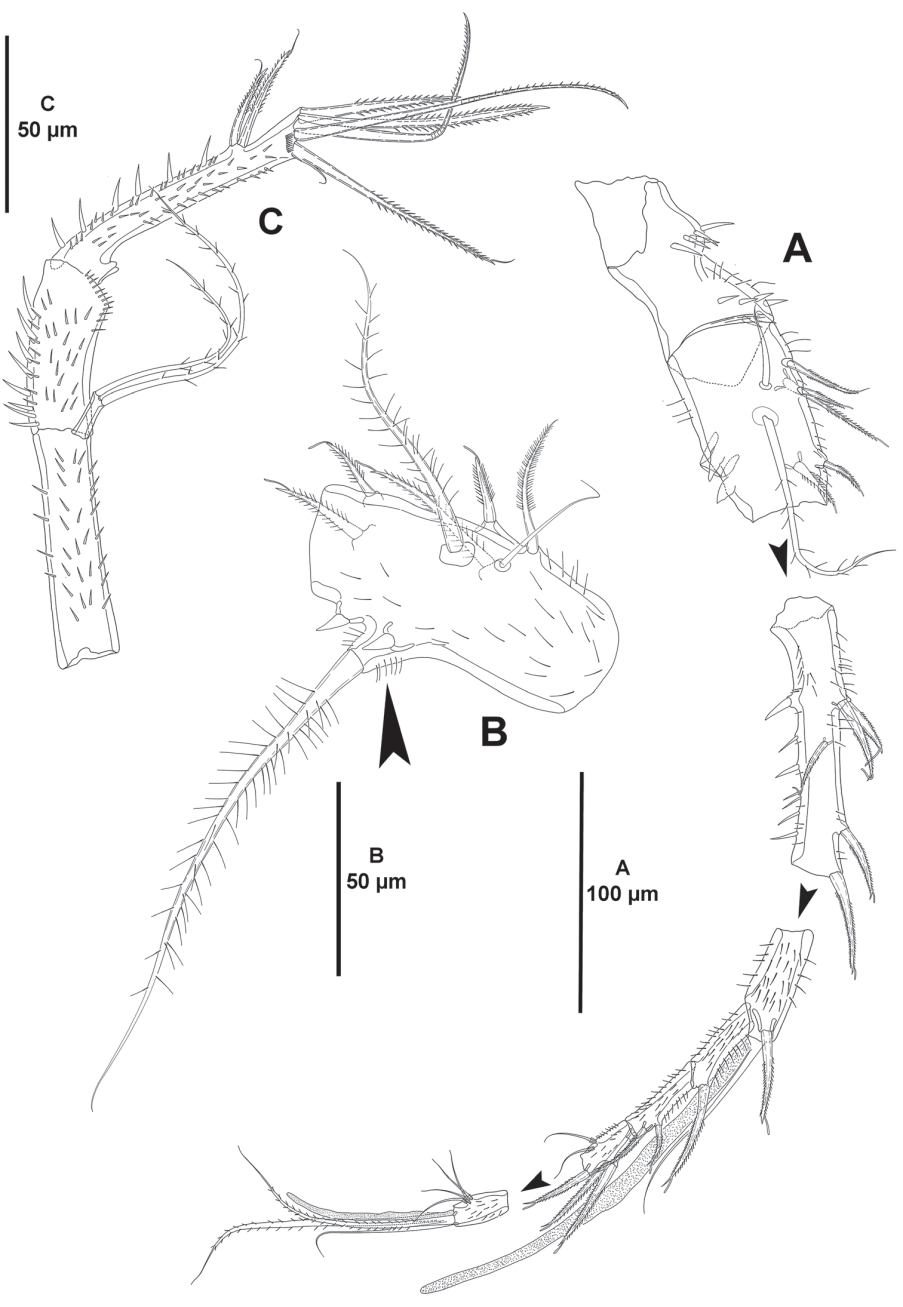
*Mesocletodes brevisetosus* sp. n., female holotype. **A** antennule **B** second segment of antennule, showing strong protrusion with strong seta **C** antenna.

*Antennule* (Fig. 4A, B) octa-segmented; all segments with small slender spinules; first segment without armature, with one medial and one distal row of strong spinules; second segment with strong protrusion bearing one strong element (arrowed in Fig. 4B), and two strong spinules; third segment elongated, as long as fourth–seventh segments combined; eighth segment smallest. Armature formula as follows: 1-[0], 2-[5sp+3se], 3-[5sp], 4-[1sp+(1se+ae)], 5-[1sp], 6[3sp], 7-[2sp+2se], 8-[5se+acro]. Spinulose, spiniform elements (sp) with STE.

*Antenna* (Fig. 4C). Basis elongated, covered with small spinules. Exopod uni-segmented, with two apical setae. Endopod bi-segmented; first segment with strong inner spinules, covered with smaller spinules, shorter than basis; second segment covered with small spinules, inner margin with stronger spinules and with two thin subdistal spines with STE, and six apical elements (one inner strong spinulose spine, one spinulose spine, two geniculate spinulose elements, and two outer elements fused basally of which innermost longer and with STE).

**Figure 5.**
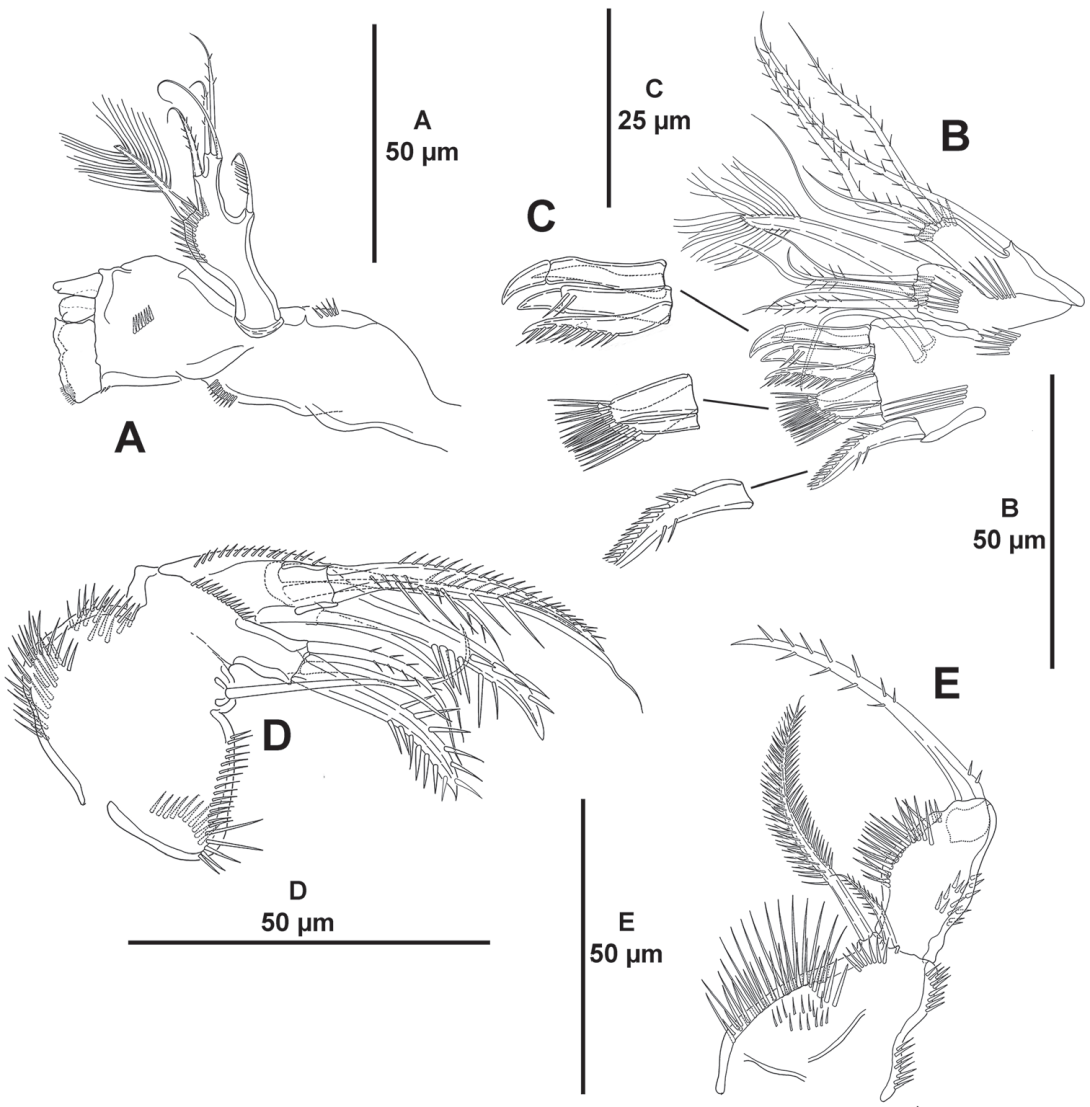
*Mesocletodes brevisetosus* sp. n., female holotype. **A** mandible **B** maxillule **C** distal armature of the arthrite of the maxillule **D** maxilla **E** maxilliped.

*Mandible* (Fig. 5A). Mandibular gnathobase with some surface spinules, with two distal blunt teeth, and with broad grinding face. Palp uni-segmented (exopod and endopod fused to basis), with one basal, one exopodal, and one subdistal and three apical endopodal setae.

*Maxillule* (Fig. 5B, C). Praecoxal arthrite with some very long spinules, two surface setae, and eight apical spines (Fig. 5C). Coxa with five elements, strongest fused to coxa. Basis with subapical row of spinules and five setae.

*Maxilla* (Fig. 5D). Syncoxa with outer and inner spinules, with smaller spinules close to allobasis; with two endites; proximal endite with one slender seta, distal endite with one strong spinulose element, one pinnate and one bare seta. Allobasis with longitudinal row of outer spinules, with one strong spinulose spine fused to allobasis, one slender seta and one spinulose spine. Endopod uni-segmented, very small, with two setae.

*Maxilliped* (Fig. 5E) subchelate, strong. Syncoxa with inner long and outer small spinules as shown, with two setae, one of which strong and longer than syncoxa. Basis unarmed, with inner long and outer small spinules. Endopod uni-segmented, fused to strong spinulose claw.

**Figure 6.**
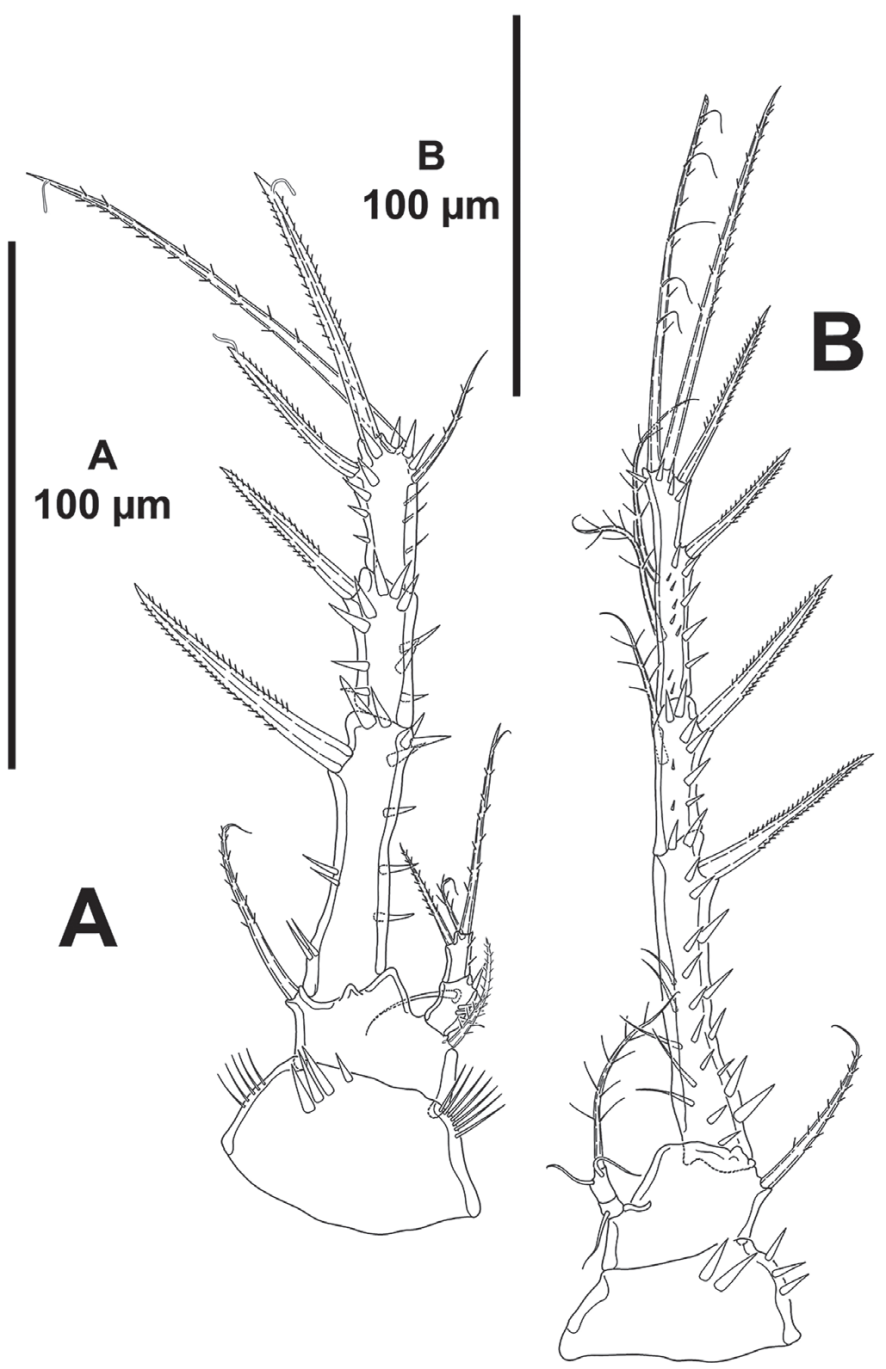
*Mesocletodes brevisetosus* sp. n., female holotype. **A** P1, anterior **B** P2, anterior.

*P1* (Fig. 6A). Coxa with outer and inner slender spinules, with stronger spinules medially. Basis seemingly without spinular ornamentation, with outer and inner spine, the former longer. Exopod tri-segmented; EXP1 as long as following segments combined; with spinules as depicted; EXP1 and EXP2 without inner armature; EXP3 with four elements, of which outer and apical elements with STE, inner seta slender and reduced. Endopod bi-segmented, small; ENP1 with few inner spinules and one inner seta; ENP2 with few inner spinules, two apical and one outer seta.

**Figure 7.**
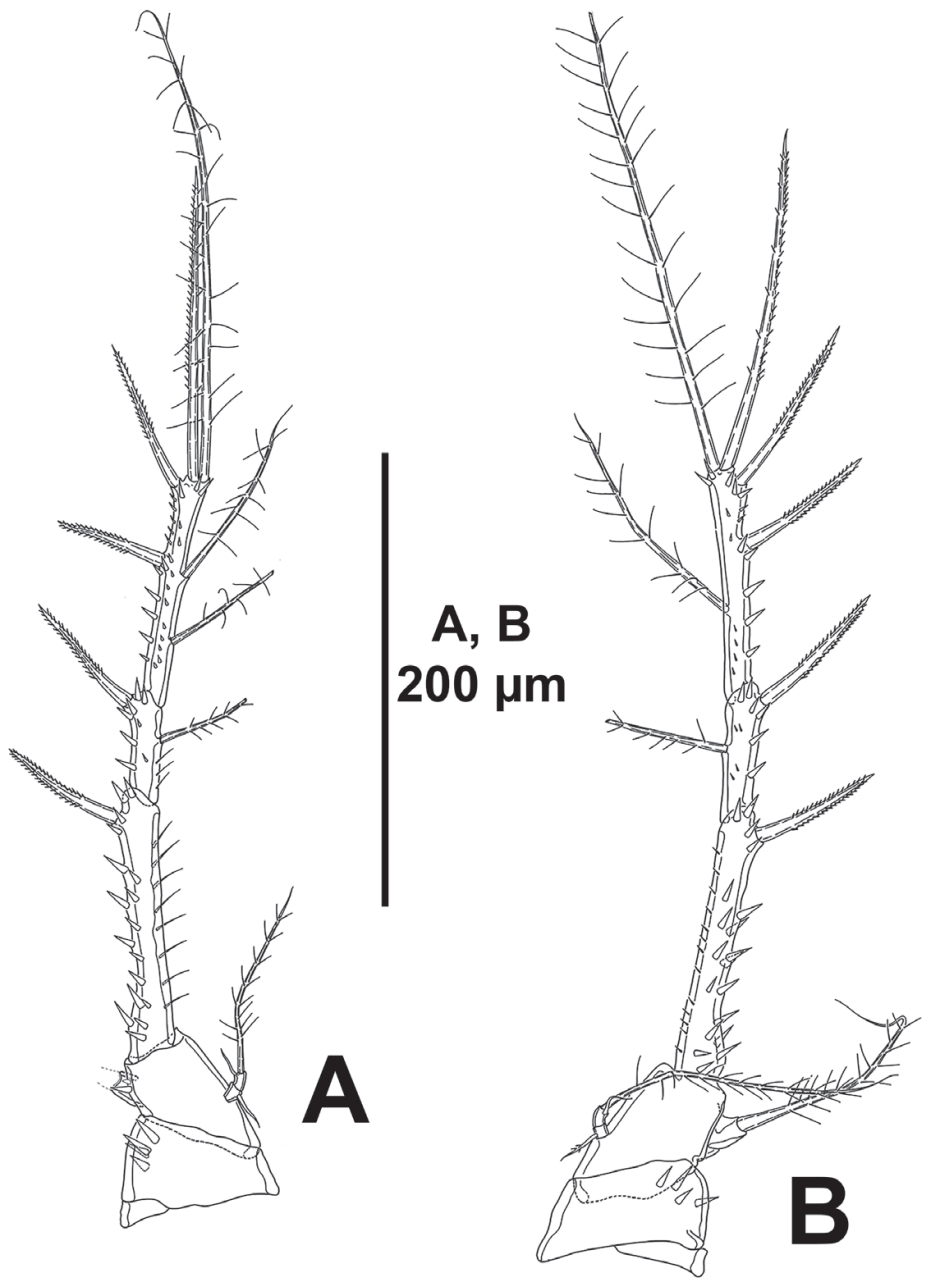
*Mesocletodes brevisetosus* sp. n., female holotype. **A** P3, anterior **B** P4, anterior.

*P2–P4* (Figs 6B, 7A–B). Praecoxa as in P3 and P4, small (see Fig. 7A, B). Coxa with few strong spinules close to outer distal corner. Basis seemingly without spinules (but two small spinules were observed at the base of the basal setophore of P3); outer basal seta of P2 spiniform, without setophore (Fig. 6B), of P3 and P4 setiform, long, with well-developed setophore (Fig. 7A, B). Exopod tri-segmented; EXP1 and EXP3 elongated, subequal in length, EXP2 shortest; EXP1 without, EXP2 with inner seta; P2 EXP3 and P3 EXP3 with two outer spines, two apical elements, and two inner setae (Fig. 6A–B), P4 EXP3 with two outer spines, two apical elements and one inner seta (Fig. 7B). Endopod bi-segmented; first segment with one inner seta; second segment of P2 with one inner and two apical setae (Fig. 6B), of P3 (Fig. 7A) and P4 (Fig. 7B) with one inner and one apical seta.

**Figure 8.**
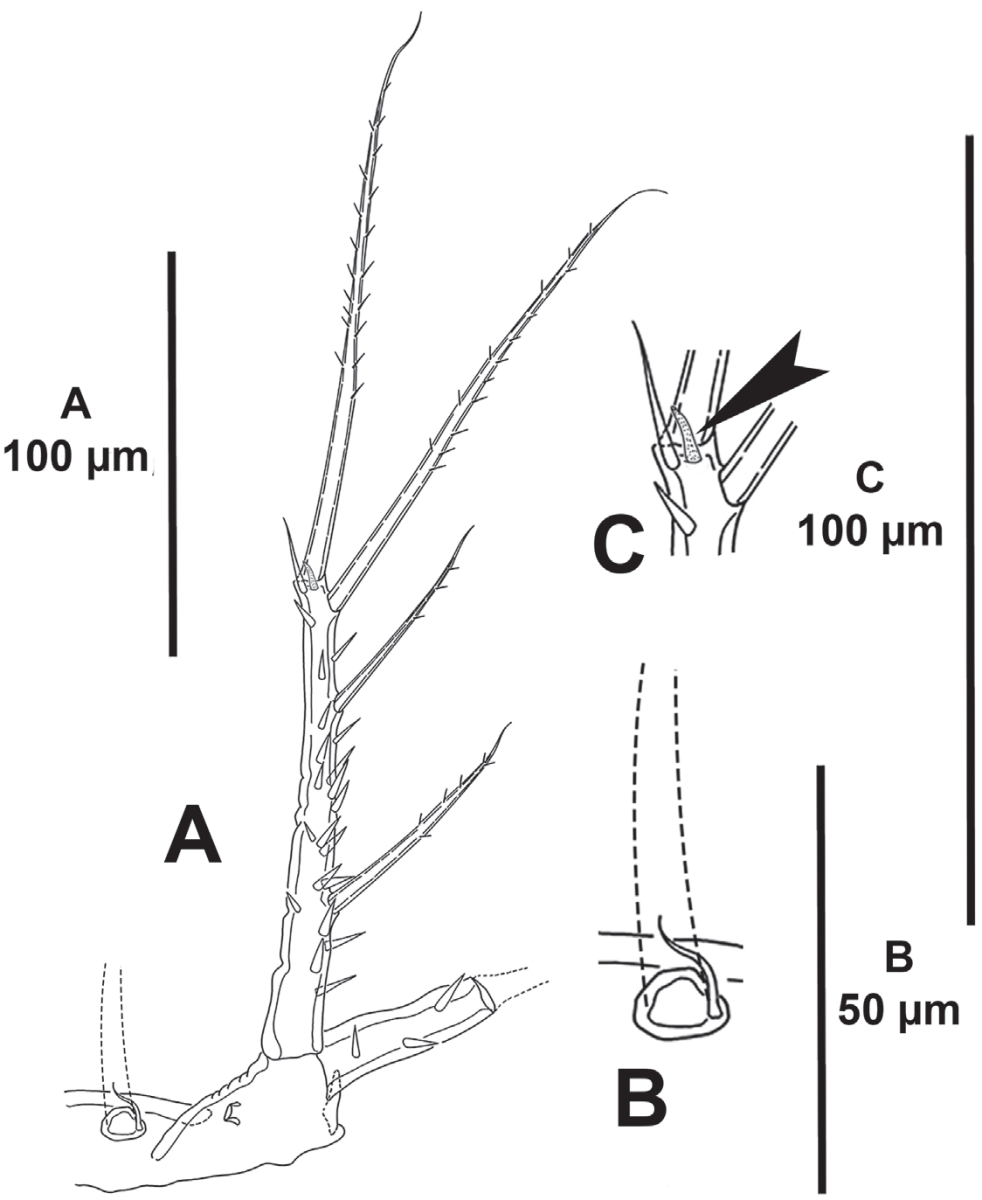
*Mesocletodes brevisetosus* sp. n., female holotype. **A** P5, anterior **B** endopodal setae **C** distal part of exopod, showing apical tube-pore.

*P5* (Fig. 8A–C) with some spinules on baseoendopodal setophore. Endopodal lobe poorly developed, with two setae (innermost lost during dissection), of which outermost very small (Fig. 8A, B). Exopod distinct, long, slender, 7.8 times as long as wide (maximum width measured at its base), with spinules as figured, with three outer, one apical and one inner seta, and one subdistal tube pore (arrowed in Fig. 8C).

Armature formula as follows:

**Table:** 

	EXP	ENP
P1	0.0.121	1.021
P2	0.1.222	1.120
P3	0.1.222	1.020
P4	0.1.122	1.020
P5	320+subdistal tube pore	2

###### Description of male.

Unknown.

###### Etymology.

The specific epithet is derived from the Latin adjective *brevis*, meaning short, and the Latin noun *seta*, meaning hair, and refers to the reduced innermost seta of the female P5 EXP. The name is a noun in the nominative singular.

###### Remarks.

*Mesocletodes brevisetosus* sp. n. seems to be more closely related to *M. dorsiprocessus* than to *M. bicornis*, both from the Angola Basin, by the combination of several characters: 1) serrated posterior margin of the cephalothorax and P2-bearing somite to penultimate urosomite (serrated posterior margin on cephalothorax, P2-bearing somite to first half of genital double-somite in *M. bicornis*), 2) relative length of the setae of the antennary exopod (subequal in *M. dorsiprocessus* and in *M. brevisetosus* sp. n., but one of them noticeably reduced in *M. bicornis*), 3) presence of an inner seta on P2–P4 ENP1 in *M. dorsiprocessus* and *M. brevisetosus* sp. n., but absent in *M. bicornis*, 4) number of setal complements on P1–P4 ENP2 (3, 3, 2, 2 in *M. dorsiprocessus* and *M. brevisetosus* sp. n., but 2, 4, 4, 4 in *M. bicornis*), 5) number of setae on the female P5 endopodal lobe (two setae in *M. dorsiprocessus* and *M. brevisetosus* sp. n., but three in *M. bicornis*), 6) position of the inner seta of the female P5 EXP (issuing subapically in *M. dorsiprocessus* and *M. brevisetosus* sp. n., but situated in distal third of inner margin of ramus in *M. bicornis*), and 7) degree of development of the endopodal lobe of the female P5 (without any trace of endopodal lobe in *M. dorsiprocessus* and *M. brevisetosus* sp. n., but endopodal lobe discernible in *M. bicornis*). *Mesocletodes dorsiprocessus* and *M. brevisetosus* sp. n. can be separated based on the number of setae on the endopodal lobe of the mandibular palp (one seta in *M. dorsiprocessus*, but four elements in *M. brevisetosus* sp. n.), the relative length of the innermost seta of the female P5 EXP (well-developed in *M. dorsiprocessus*, but very reduced in *M. brevisetosus* sp. n.), appearance of the cuticula (plain in *M. dorsiprocessus*, but reticulated in *M. brevisetosus* sp. n.), armature complement of the seventh antennular segment (six in *M. dorsiprocessus*, but four in *M. brevisetosus* sp. n.), and number of setae on the syncoxa of the maxilliped (one in *M. dorsiprocessus*, but two in *M. brevisetosus* sp. n.).

##### Mesocletodes simplex

Taxon classificationAnimaliaHarpacticoidaArgestidae

sp. n.

http://zoobank.org/536B7890-8AB1-4FA5-884C-34A5C5895D5E

###### Material examined.

One dissected female holotype mounted onto five slides (ICML-EMUCOP-130207-01); Talud X cruise; February 13, 2007; coll. S. Gómez.

###### Type locality.

Southern Trough of Guaymas Basin, Gulf of California, México (27°01'N, 110°53’04"W), 1642 m depth (see Fig. 1); coll. S. Gómez.

###### Diagnosis (based on the female only).

Body subcylindrical. Cephalothorax with dorsal cuticular process curved posteriorly. Genital somite and third urosomite incompletely fused dorsolaterally. Anal somite quadrate from dorsal view, with simple dorsal cuticular process curved posteriorly. Caudal rami subcylindrical, 2.5 times as long as wide, with seven setae. Antennule octa-segmented, second segment with protrusion bearing a long seta, but not as pronounced as in other species of the genus. Antenna with basis and uni-segmented exopod. Gnathobase of mandible with grinding face, and tri-segmented palp. Maxillary syncoxa with two endites, proximal endite with one, distal endite with three elements; endopod uni-segmented, with two setae. Syncoxa of maxilliped with one seta. P4 ENP2 with four setae. Outer setae of the P5 EXP issuing close to each other.

###### Description of female.

**Figure 9.**
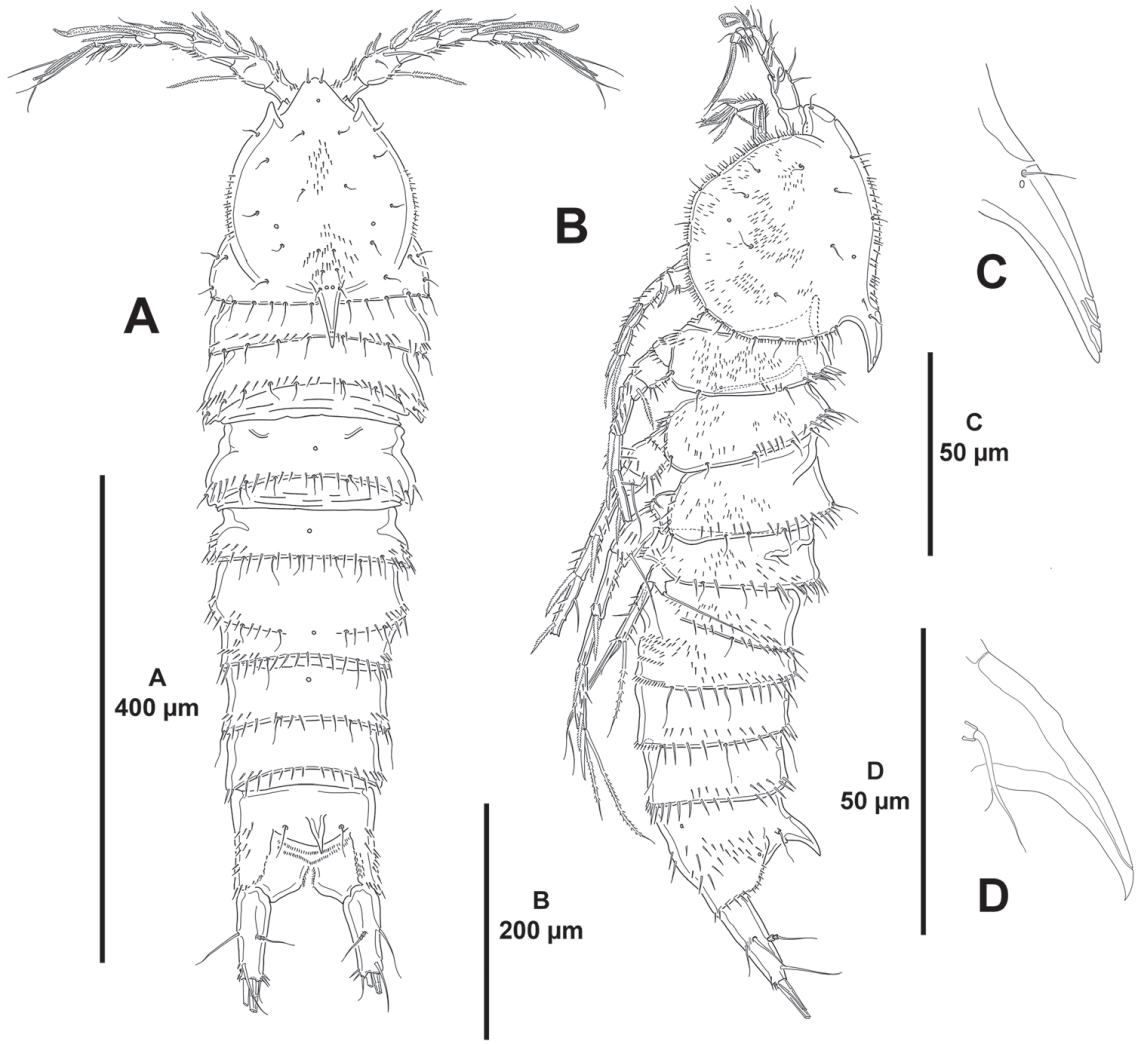
*Mesocletodes simplex* sp. n., female holotype. **A** habitus, dorsal **B** habitus, lateral **C** dorsal cuticular process of cephalothorax showing cuticular ornamentation **D** dorsal cuticular process of anal somite showing cuticular ornamentation.

**Figure 10.**
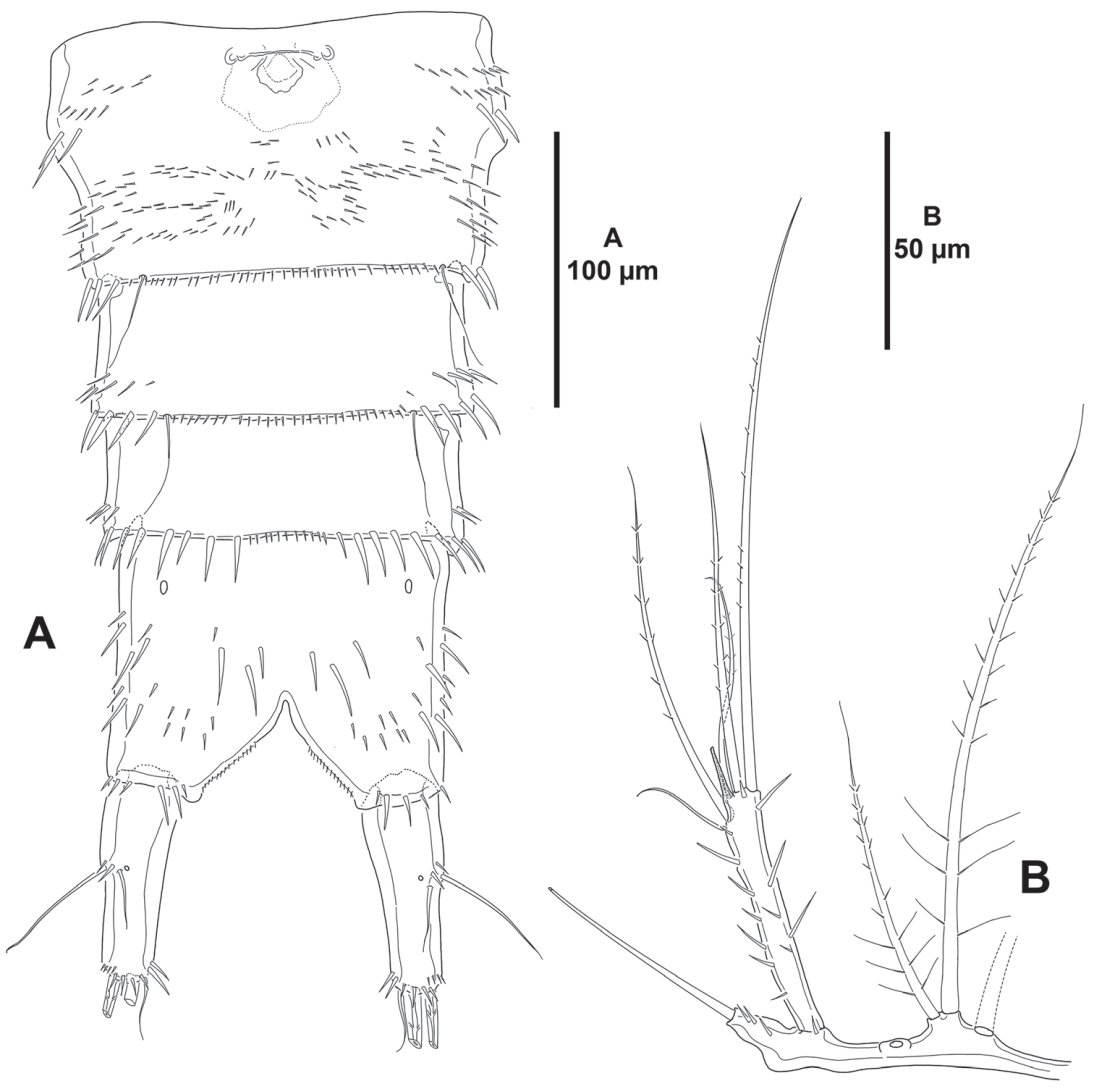
*Mesocletodes simplex* sp. n., female holotype. **A** urosome, ventral, P5-bearing somite omitted **B** P5, anterior.

*Body*: total length 725 µm measured from anterior margin of rostrum to posterior margin of caudal rami, subcylindrical, tapering slightly posteriorly, without clear demarcation between prosome and urosome (Fig. 9A, B). Rostrum fused to cephalothorax, with two sensilla. Cephalothorax 0.24 times as long as entire body length; ornamented with sensilla and spinular patches as shown; with dorsal cuticular process curved posteriorly, the latter as in Fig. 9C. P2–P5-bearing somites with sensilla and small spinules along posterior margin, with minute spinules laterally. Genital somite and third urosomite (genital double-somite) incompletely fused dorsolaterally (Fig. 9A, B) (i.e. posterior margin of genital somite indicated by suture with transverse row of spinules and few sensilla dorsolaterally), completely fused ventrally (Fig. 10A); first half of genital double-somite with medial genital field proximally (Fig. 10A), with few minute spinules close to lateral margins, second half with more dense spinular patches ventrally as shown and with minute spinules along posterior margin between pair of long ventral sensilla. Fourth urosomite with short row of small spinules laterally (Fig. 9B), ventrally with transverse row of larger spinules along posterior margin interrupted by longitudinal row of minute spinules along posterior margin flanked by two long sensilla (Fig. 10A). Fifth urosomite as preceding somite except for shorter transverse row of minute spinules ventrally, without sensilla (Fig. 10A).

**Figure 11.**
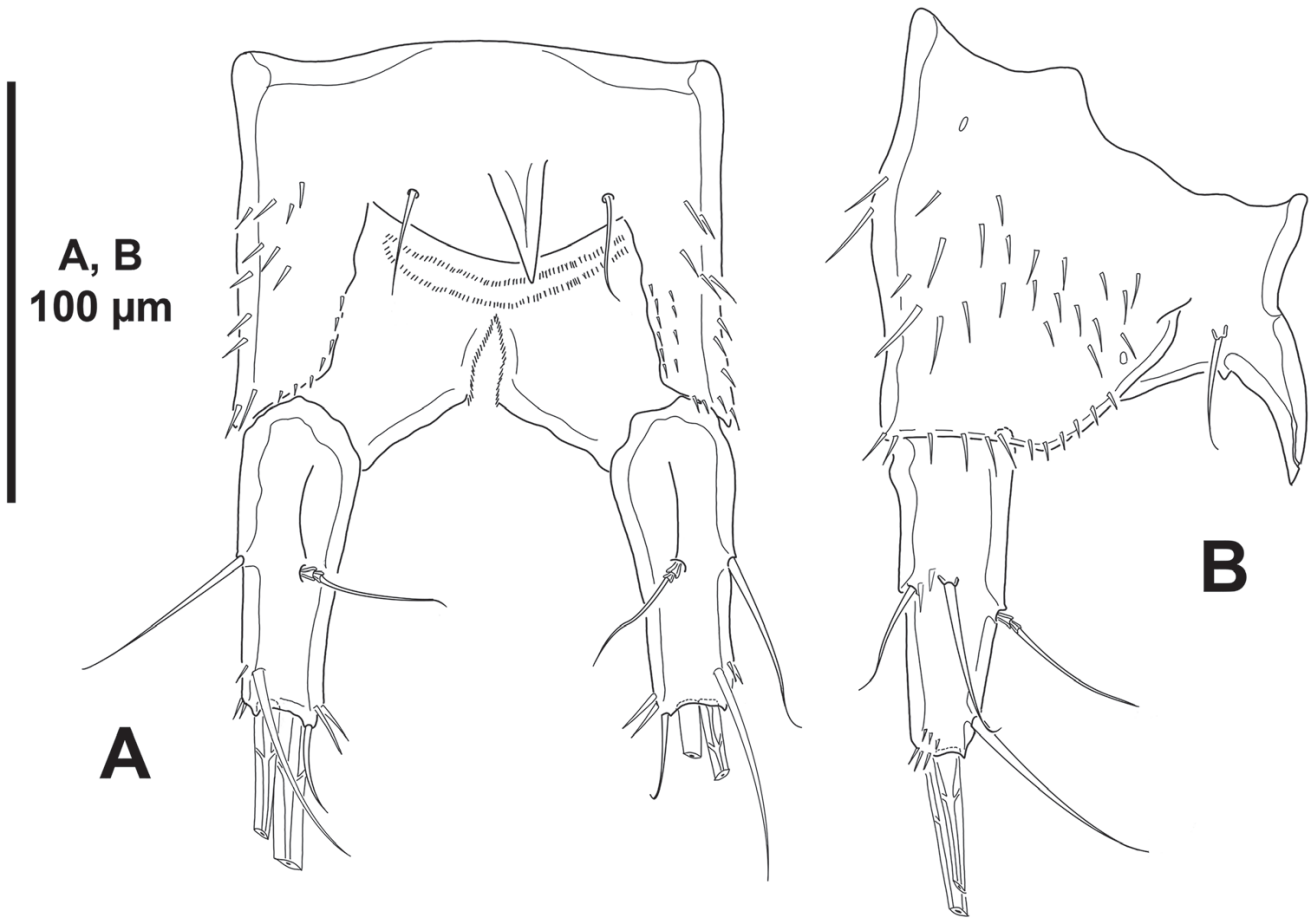
*Mesocletodes simplex* sp. n., female holotype. **A** anal somite, dorsal **B** anal somite, lateral.

*Anal somite* (Figs 9A, B, 10A, 11A, B) quadrate from dorsal view, nearly as long as two preceding somites combined; posterior margin cleft medially; anal operculum rounded and smooth, flanked by two sensilla; with dorsal (Figs 9A, 11A), lateral (Figs 9B, 11B) and ventral (Fig. 10A) spinules as figured; with simple dorsal cuticular process curved posteriorly (Figs 9A, B, D, 11A, B), the latter with a tiny aperture (Figs 9D, 11B).

*Caudal rami* (Figs 9A, B, 10A, 11A, B) subcylindrical, slightly tapering posteriorly, nearly as long as anal somite and 2.5 times as long as wide; with seven setae as follows: setae I and II located midway lateral margin, the former ventral to the latter and shorter; seta III as long as seta II, arising close to outer distal corner; setae IV and V situated distally; seta VI smallest, arising at inner distal corner; dorsal seta VII bi-articulated, somewhat shorter than seta II.

**Figure 12.**
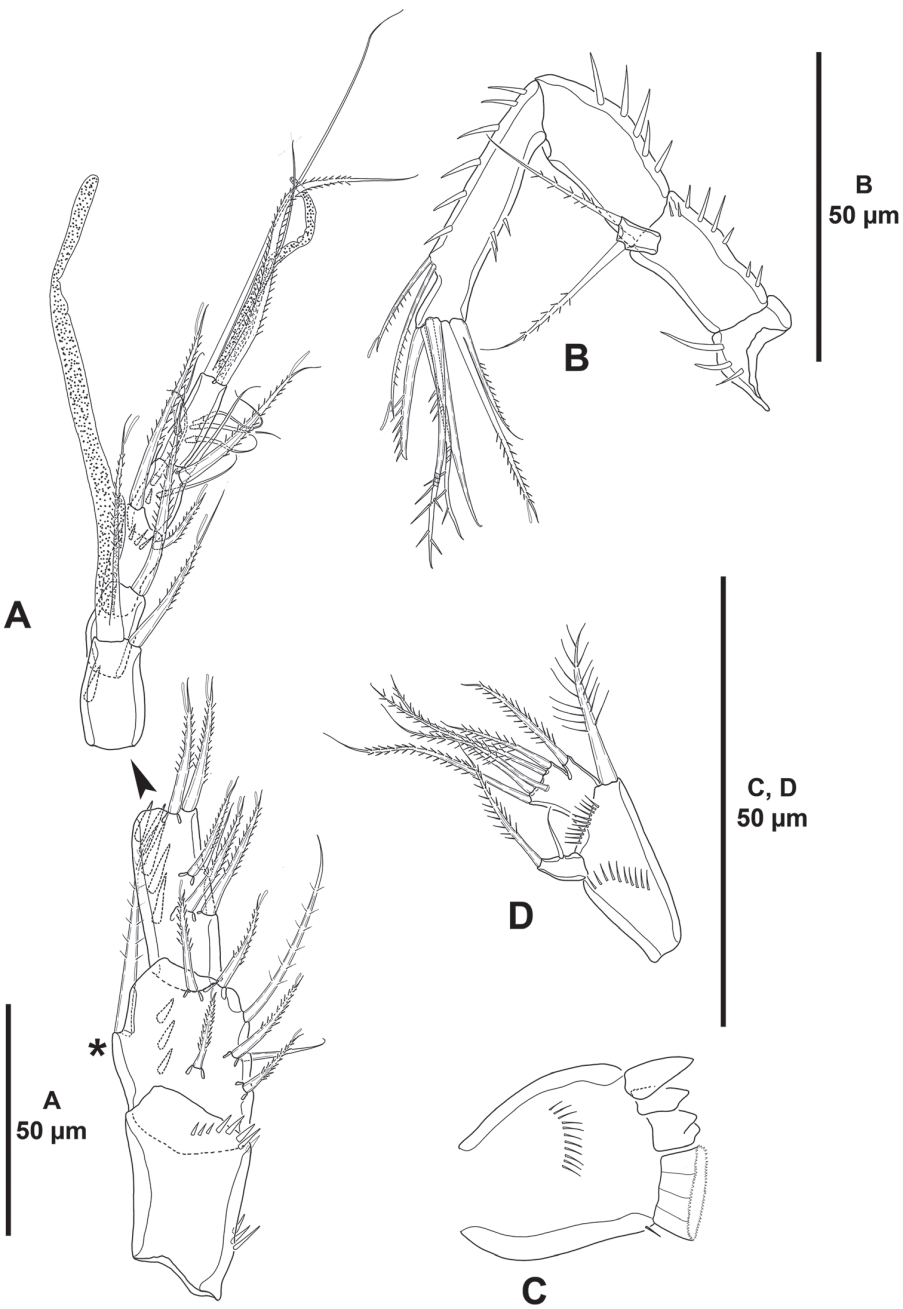
*Mesocletodes simplex* sp. n., female holotype. **A** antennule, protrusion on second segment bearing a strong seta indicated by an asterisk **B** antenna **C** mandibular gnathobase **D** mandibular palp.

*Antennule* (Fig. 12A) octa-segmented; first segment with one proximal and one subdistal row of spinules; second and third segments with longitudinal short row of strong spinules; second segment somewhat globose and with protrusion bearing a long seta not as pronounced as in other species of the genus (indicated by an asterisk in Fig. 12A); third segment two times as long as wide; fourth segment with one, fifth segment without spinules; sixth segment with short transverse row of smaller spinules; seventh segment with one, eighth segment without spinules. Armature formula as follows: 1-[0], 2-[5sp+3se], 3-[5sp+1se], 4-[1sp+(1sp+ae)], 5-[1sp], 6[2sp], 7-[1sp+3se], 8-[5se+acro]. Spinose, spiniform elements (sp) with STE.

*Antenna* (Fig. 12B). Coxa small, with few strong spinules. Basis with inner spinules. Exopod uni-segmented, with two setae. Endopod bi-segmented; first segment with strong inner spinules; second segment with some outer small spinules, inner margin with strong spinules and two thin lateral spines with STE, and six apical elements (one inner strong spinulose spine, two geniculate spinulose and one bare element, and two outer elements fused basally of which innermost longer and with STE).

*Mandible* (Fig. 12C, D) with robust coxa. Gnathobase with row of surface spinules, three distal single teeth as shown and several fused tooth-like elements forming a broad grinding face (Fig. 12C). Palp tri-segmented (Fig. 12D); basis with medial and distal small spinules, with one strong seta; exopod uni-segmented, small, with two setae of which innermost smaller; endopod quadrate, with six setae as shown.

**Figure 13.**
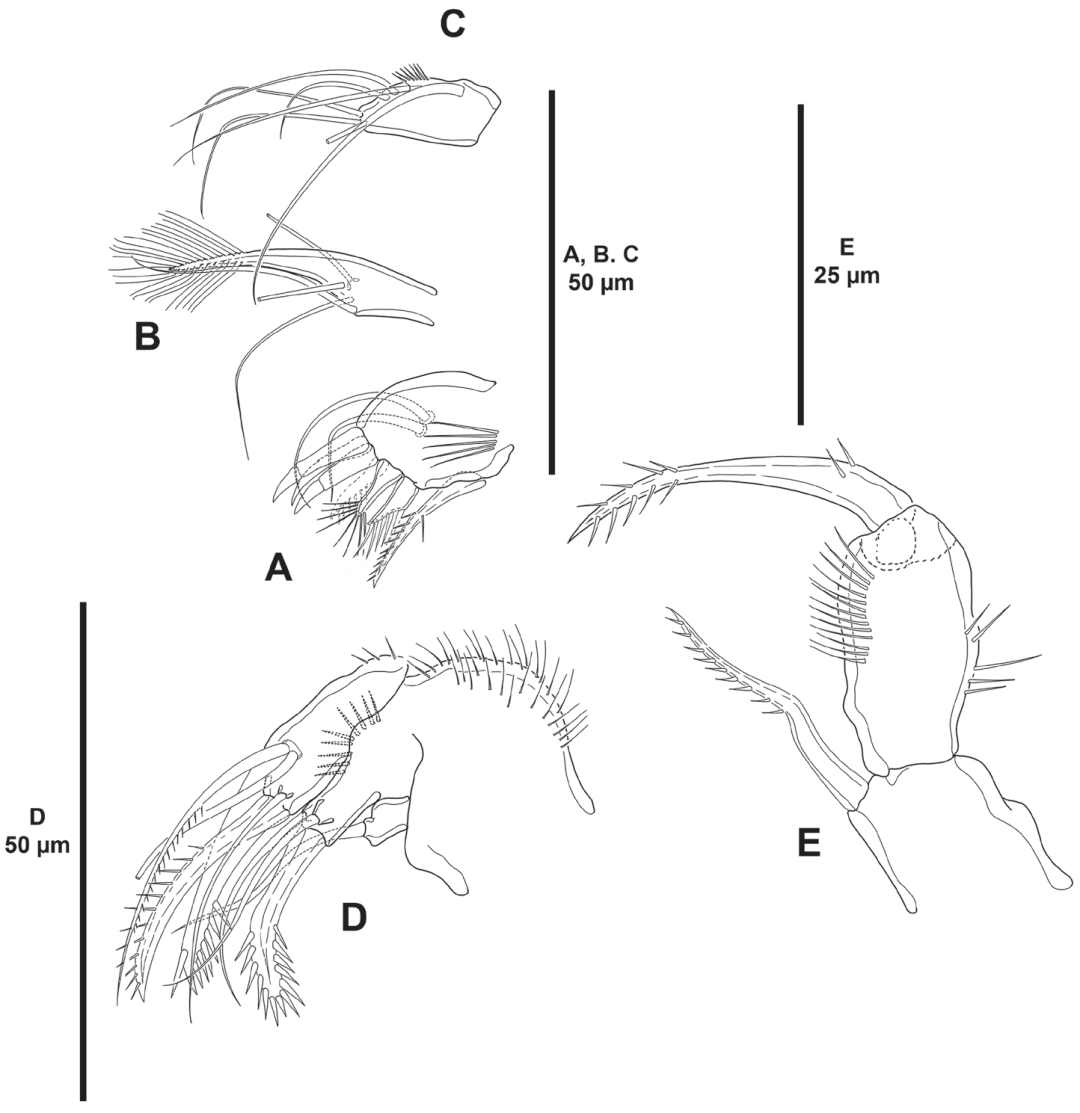
*Mesocletodes simplex* sp. n., female holotype. **A** praecoxal arthrite of maxillule **B** coxal endite of maxillule **C** basis of maxillule **D** maxilla **E** maxilliped.

*Maxillule* (Fig. 13A, B, C). Praecoxal arthrite with some very long spinules, two surface setae, and seven apical spines as shown (Fig. 13A). Coxa with five elements, strongest fused to coxa (Fig. 13B). Basis with seven setae (Fig. 13C).

*Maxilla* (Fig. 13D). Syncoxa with slender outer spinules, and with comparatively stronger spinules close to allobasis; with two endites; proximal endite with one slender seta, distal endite with one strong spinulose element and two slender smooth setae. Allobasis with few outer spinules, with one strong spinulose spine fused to allobasis, one slender seta and one spinulose spine. Endopod uni-segmented, very small; with two setae.

*Maxilliped* (Fig. 13E) subchelate, strong. Syncoxa with one spinulose strong seta slightly longer than basis; the latter with outer and inner spinules as shown. Endopod uni-segmented, fused to strong spinulose claw.

**Figure 14.**
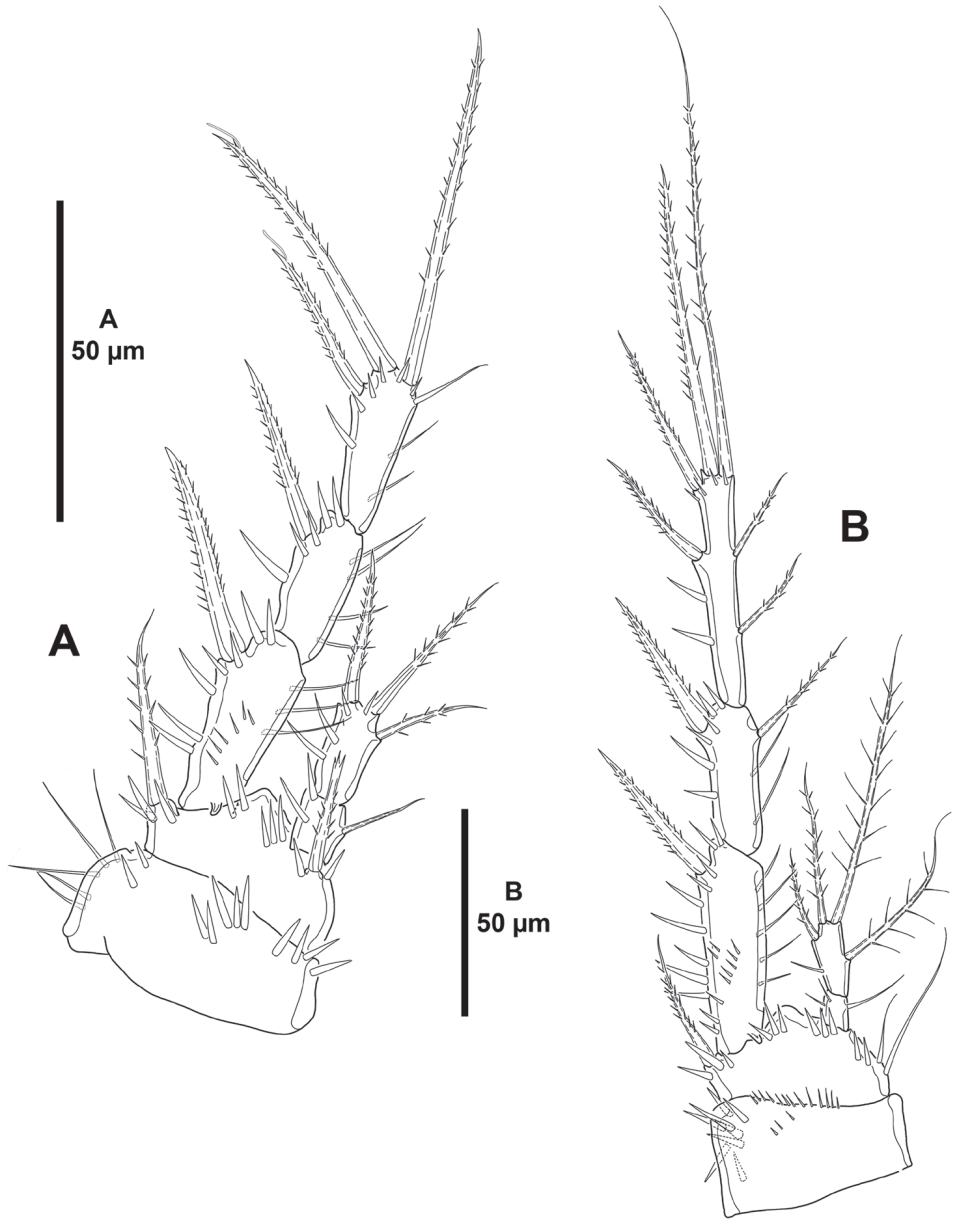
*Mesocletodes simplex* sp. n., female holotype. **A** P1, anterior **B** P2, anterior.

*P1* (Fig. 14A). Coxa with sets of outer, medial, and inner strong spinules, and with outer long slender spinules. Basis with strong spinules at base of outer and inner spine. Exopod tri-segmented; exopodal segments subequal in length, with spinules as depicted; EXP1 and EXP2 without inner armature; EXP3 with four elements, of which two outermost spines with STE, innermost slender and reduced. Endopod bi-segmented, not reaching tip of EXP1; ENP1 with few outer spinules and one inner seta, shorter than ENP2; the latter with few outer spinules and three elements.

**Figure 15.**
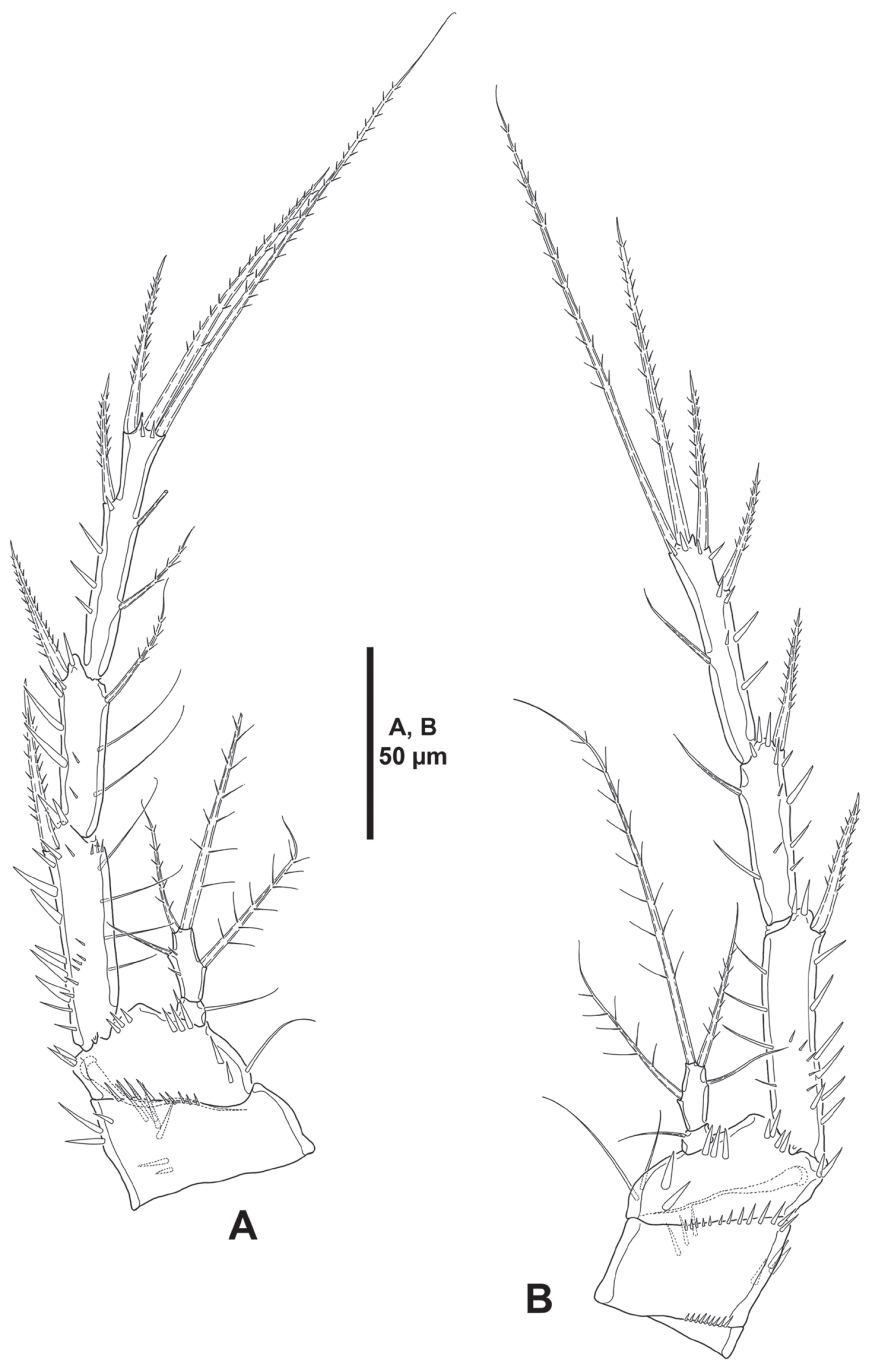
*Mesocletodes simplex* sp. n., female holotype. **A** P3, anterior **B** P4, anterior.

*P2–P4* (Figs 14B, 15A, B). Praecoxa as in P4 (see Fig. 15B), small, with transverse row of small spinules close to coxa. Coxa with small spinules close to basis, and with stronger outer spinules on anterior face and some medial strong spinules on posterior face. Basis with strong spinules at base of outer element, between rami, and at base of endopod, and with slender long inner spinules; basis of P2 (Fig. 14B) with outer spiniform element, of P3 and P4 (Fig. 15A, B) with outer slender bare seta. Exopod tri-segmented; segments slender and elongate, ornamented as shown; EXP1 without, EXP2 with inner seta; P2 EXP3 and P3 EXP3 with two outer spines, two apical elements, and two inner setae (Figs 14B, 15A), P4 EXP3 (Fig. 15B) with two outer spines, two apical elements and one inner seta.

*P5* (Fig. 10B) with some spinules on baseoendopodal setophore; with long outer basal seta. Endopodal lobe poorly developed, with three setae (innermost lost during dissection), of which outermost and medial elements close to each other, innermost separated from the former two elements. Exopod distinct, long, slender, 6.7 times as long as wide (maximum width at distal part), with outer and inner spinules as figured, with three outer and two apical setae, and one outer distal tube pore.

Armature formula as follows:

**Table:** 

	EXP	ENP
P1	0.0.121	1.120
P2	0.1.222	1.121
P3	0.1.222	1.121
P4	0.1.122	1.121
P5	320+distal tube pore	3

###### Description of male.

Unknown.

###### Etymology.

The specific epithet is derived from the Latin adjective *simplex*, meaning simple, and refers to the simple (not bifurcated) dorsal process on the anal somite. The name is an adjective in the nominative singular.

###### Remarks.

*Mesocletodes simplex* sp. n. is attributed here to Bodin’s (1968) *abyssicola* group. The dorsal process on the anal somite is simple in most species within this genus, but it is bifurcated in *M. brevifurca*, *M. katharinae* Soyer, 1964, *M. meteorensis*, *M. monensis*, and *M. soyeri* Bodin, 1968. The appendages of the species of this group exhibit an amalgam of conditions, most of which are shared with some species of the *inermis* group *sensu* Bodin (1968). The antenna of most species of Bodin’s (1968) *abyssicola* group possesses a basis, but the condition of the antenna is inconclusive for *M. bathybia* Por, 1964a and *M. brevifurca*. Also, the antennary exopod of most species of this group is uni-segmented with two setae, but *M. abyssicola* seems to be unique among these species in that it is represented by one seta only (the condition of the antennary exopod of *M. monensis* is inconclusive, and the exopod of *M. bathybia* remains unknown). The mandibular palp of most species of this group is bi-segmented (exopod incorporated to basis, endopod uni-segmented), but uniramous in *M. soyeri*, *M. bathybia* and *M. katharinae*, and tri-segmented (with basis, uni-segmented exopod, and uni-segmented endopod) in *M. simplex* sp. n. The palp of the maxillule is uni-segmented in *M. abyssicola*, *M. katharinae*, *M. meteorensis*, *M. robustus* Por, 1965, *M. simplex* sp. n., and *M. soyeri*. Also, the proximal and distal endites of the maxilla possess one and three setae, respectively, in *M. katharinae*, *M. meteorensis*, *M. simplex* sp. n., and *M. robustus*, but two setae only in *M. soyeri*. The syncoxa of the maxilliped possesses two setae in most species, but one seta only in *M. brevifurca*, *M. simplex* sp. n., and *M. abyssicola* (the maxilliped of *M. monensis*, *M. bathybia*, and *M. dolichurus* Smirnov, 1946 remains unknown). The P1 ENP is uni-segmented with three setae in *M. abyssicola*, *M. robustus*, and *M. soyeri*, but uni-segmented with one seta only in *M. bathybia*. A uni-segmented P1 ENP is present also in the species of Bodin’s (1968) *inermis* group (e.g. *M. makarovi* Smirnov, 1946, *M. guillei* Soyer, 1964, *M. inermis*, *M. trisetosa* Schriever, 1983, and *M. quadrispinosa*). The P1 ENP is bi-segmented with an armature complement of 0,3 in the first and second segment, respectively, in *M. brevifurca*, *M. dolichurus* and *M. katharinae*, and 1,3 in *M. meteorensis* and *M. simplex* sp. n. The P2–P4 ENP is uni-segmented in *M. monensis*, *M. dolichurus*, *M. robustus* and *M. soyeri*, and probably also in *M. abyssicola* and *M. bathybia*, but bi-segmented in the other species of the group of which, only *M. katharinae* lack the inner seta on P2 ENP1. The female P5 EXP and endopodal lobe possess five and three setae, respectively, in all the species of this group, except for *M. abyssicola* and *M. soyeri* (EXP with four, endopodal lobe with two setae). Similar armature complements and structure of P1–P5 is present in several species of the *inermis* group *sensu* Bodin (1968). The caudal rami are more than 10 times as long as wide in most species, but 2.5 to 3 times as long as wide in *M. brevifurca*, *M. meteorensis*, and *M. simplex* sp. n., and 6 times as long as wide in *M. katharinae*. *Mesocletodes simplex* sp. n. shares the relatively short caudal rami with *M. brevifurca* and *M. meteorensis*. The former can be easily separated from the latter two species by the dorsal process on the anal somite (simple in *M. simplex* sp. n., but bifurcated in the other two species). Additionally, *M. simplex* sp. n. seems to be more closely related to *M. meteorensis* than to *M. brevifurca* by the relative position of the outer setae of the female P5 EXP (both setae separated by a wide gap in *M. brevifurca*, but both setae issuing close one of each other in *M. simplex* sp. n. and *M. meteorensis*), and number of setae on the P4 ENP2 (three setae in *M. brevifurca*, but four setae in *M. simplex* sp. n. and *M. meteorensis*).

##### Mesocletodes unisetosus

Taxon classificationAnimaliaHarpacticoidaArgestidae

sp. n.

http://zoobank.org/CB4BE678-C76F-4D35-A3BD-300B3324F960

###### Material examined.

One male holotype preserved in alcohol (ICML-EMUCOP-270800-04), one male paratype preserved in alcohol (ICML-EMUCOP-270800-03), and one male paratype dissected and mounted onto seven slides (ICML-EMUCOP-270800-05); Talud IV cruise; August 27, 2000; coll. S. Gómez.

###### Type locality.

Southern Carmen Basin, Gulf of California, México (25°54.7'N, 110°11'W), 2018 m depth (see Fig. 1); coll. S. Gómez.

###### Dignosis (based on the male only).

Body subcylindrical. Cephalothorax, free prosomites and urosomites, except for anal somite, with posterior margin serrated. Cephalothorax dorsoventrally flattened, without dorsal cuticular process. Anal somite quadrate, with dorsal cuticular process. Caudal rami 14 times as long as wide, with seven elements. Antennule octa-segmented, haplocer, second segment with strong protrusion bearing one strong seta and two strong spinules. Antenna with basis, without exopod. Mandibles, maxillules, maxillae and maxillipeds strongly atrophied, nontraceable. P2 and P3 ENP1 with one inner seta. P5 endopodal lobe with one seta.

###### Description of male.

**Figure 16.**
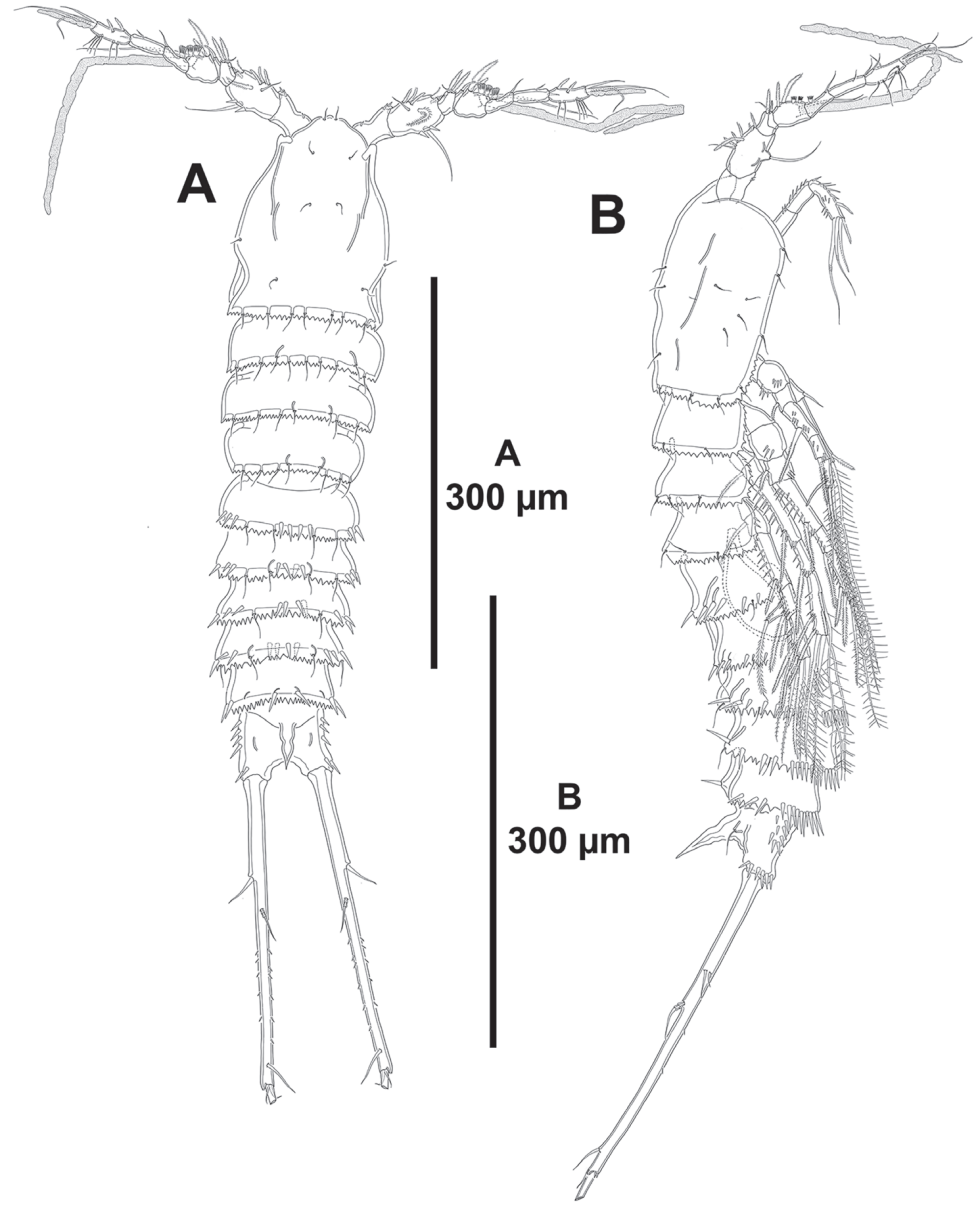
*Mesocletodes unisetosus* sp. n., male holotype. **A** habitus, dorsal **B** habitus, lateral.

**Figure 17.**
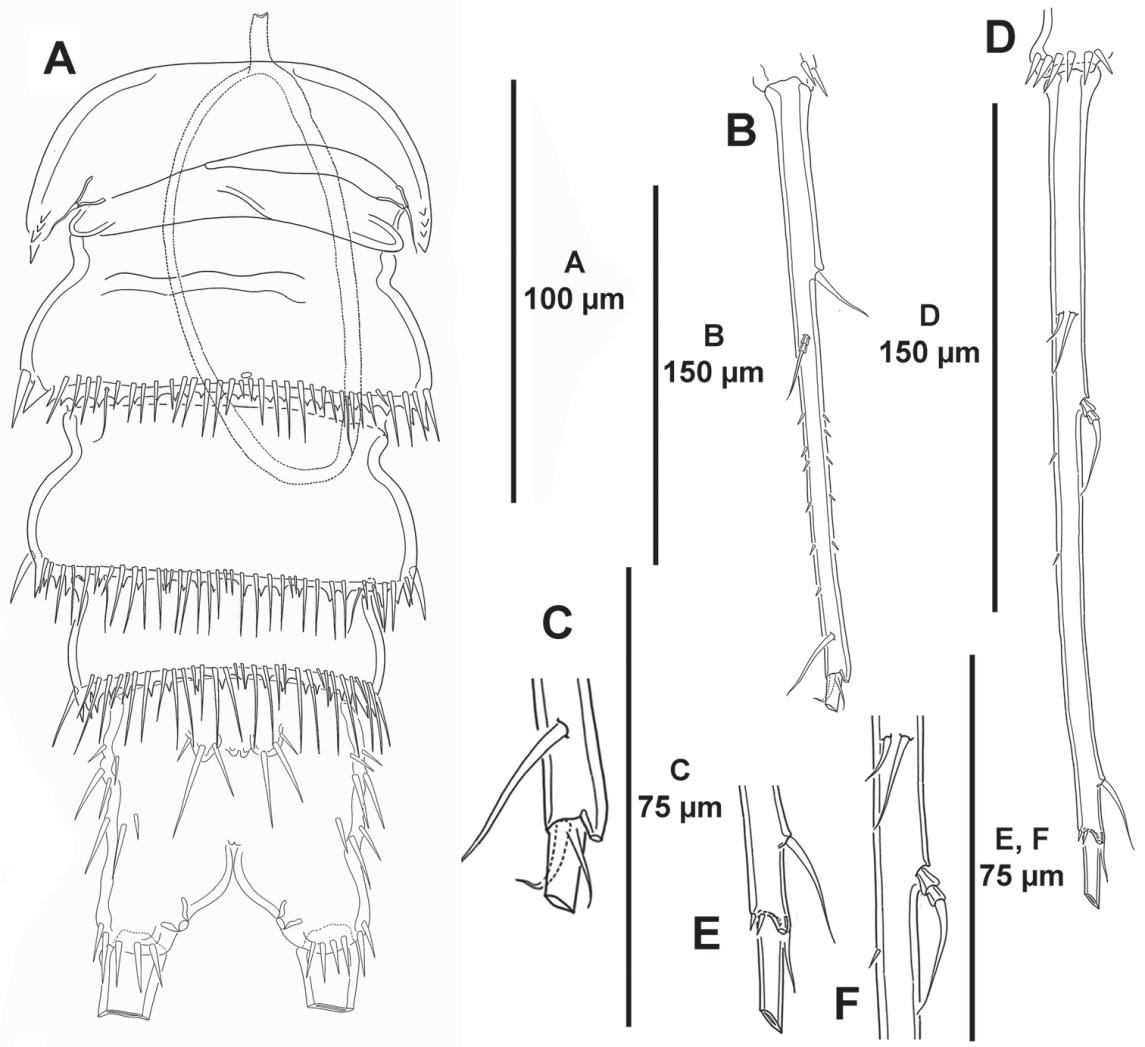
*Mesocletodes unisetosus* sp. n., male paratype. **A** urosome, ventral, P5-bearing somite omitted **B** right caudal ramus, dorsal **C** distal part of right caudal ramus, dorsal, sowing seta III, IV, V, and VI **D** left caudal ramus, lateral **E** distal part of left caudal ramus, lateral, showing seta III, IV and V **F** medial part of left caudal ramus, lateral, showing seta I, II and VII.

*Body*: total length ranging from 655 µm to 695 µm (mean= 670 µm; n= 3) measured from anterior margin of rostrum to posterior margin of caudal rami, subcylindrical (Fig. 16A, B), tapering slightly posteriorly, without clear demarcation between prosome and urosome. Cephalothorax, free prosomites and urosomites, except for anal somite, with posterior margin serrated (Fig. 16A, B), of cephalothorax and free prosomites less pronounced, of urosomites comparatively coarser; lateral margin of cephalothorax plain (Fig. 16B). Rostrum fused to cephalothorax. The latter dorsoventrally flattened, without dorsal cuticular process, with few sensilla on surface and along posterior margin. P2–P4-bearing somites without spinular ornamentation, with sensilla along posterior margins. P5-bearing somite with four medial spinules dorsally (Fig. 16A) and some spinules laterally (Fig. 16B). Genital somite as preceding somite dorsally (Fig. 16A) and laterally (Fig. 16B); ventrally (Fig. 17A) without spinular ornamentation and with two sensilla, sixth leg represented by asymmetrical plate. Third and fourth urosomites as preceding somites dorsally and laterally (Fig. 16A, B), ventrally (Fig. 17A) with serrated posterior margin and long posterior spinules. Fifth urosomite with two medial spinules dorsally (Fig. 16A), laterally (Fig. 16B) and ventrally (Fig. 17A) as two preceding somites, without sensilla.

*Anal somite* (Figs 16A, B, 17A) quadrate; dorsally without (Fig. 16A), laterally (Fig. 16B) and ventrally (Fig. 17A) with spinules as shown; with dorsal cuticular process flanked by pair of sensilla (Fig. 16A, B).

*Caudal rami* (Figs 16A, B, 17B–F) slender, exceedingly elongated, 14 times as long as wide (maximum width measured at the base of ramus), as long as urosome, almost straight in lateral view (Figs 16B, 17D), covered with small spinules; with seven elements as follows: seta I and II issuing laterally in distal part of first third of ramus, the former ventral to and smaller than the latter (Fig. 17D, F); seta III situated subdistally on dorsal surface (Fig. 17B–E); seta IV reduced, fused basally to seta V (Fig. 17B, C, E), the latter longest; seta VI reduced, somewhat smaller than seta IV, arising at distal inner corner (Fig. 17B, C); dorsal seta VII bi-articulated, situated subdistally on posterior part of first half of ramus (Fig. 17B, D, F); with large outer pore distally (Fig. 17B–E).

**Figure 18.**
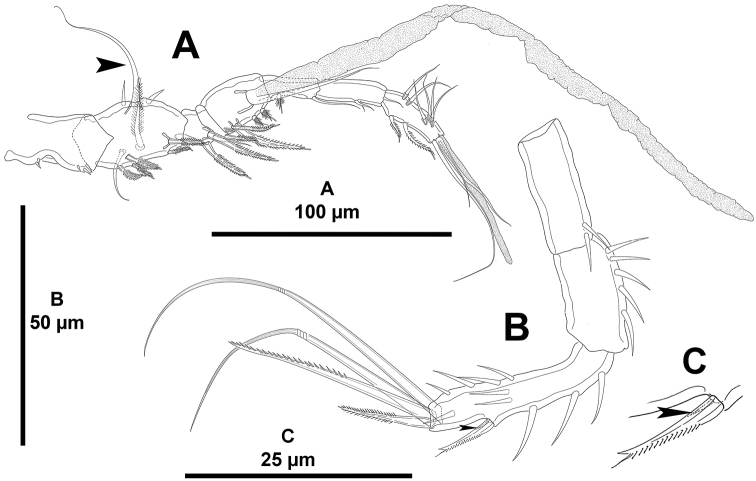
*Mesocletodes unisetosus* sp. n., male paratype. **A** antennule **B** antenna, reduced lateral spine arrowed **C** lateral armature of the antenna, reduced spine arrowed.

*Antennule* (Fig. 18A) octa-segmented, haplocer; first segment without armature, with some medial and some distal spinules; second segment with strong protrusion bearing one strong element (arrowed in Fig. 18A), with two strong spinules; third and fourth segments smallest; fifth segment somewhat swollen, with aesthetasc fused basally to slender seta, with four spiniform elements, two of which modified; sixth segment with one normal and one modified element; sixth to eight segments elongated, subequal in length. Armature formula as follows: 1-[0], 2-[6sp+2se], 3-[2sp+1se], 4-(1sp), 5-[4sp+ (1se+ae)], 6[1sp+1se], 7-[3se], 8-[2sp+7se+acro]. Spinose, spiniform elements (sp) with STE.

*Antenna* (Fig. 18B). Basis elongate, with few inner spinules on distal corner. Exopod absent. Endopod bi-segmented; first segment with strong inner spinules, as long as basis; second segment with inner strong spinules proximally and medially, with outer spinules on distal half of segment, laterally with one well-developed lateral spine with STE, and one very reduced element (the latter indicated in Fig. 18B, C), and with four apical elements (two spinulose spines, of which innermost smaller, and two geniculate elements).

*Mandibles, maxillules, maxillae and maxillipeds* strongly atrophied, non-traceable.

**Figure 19.**
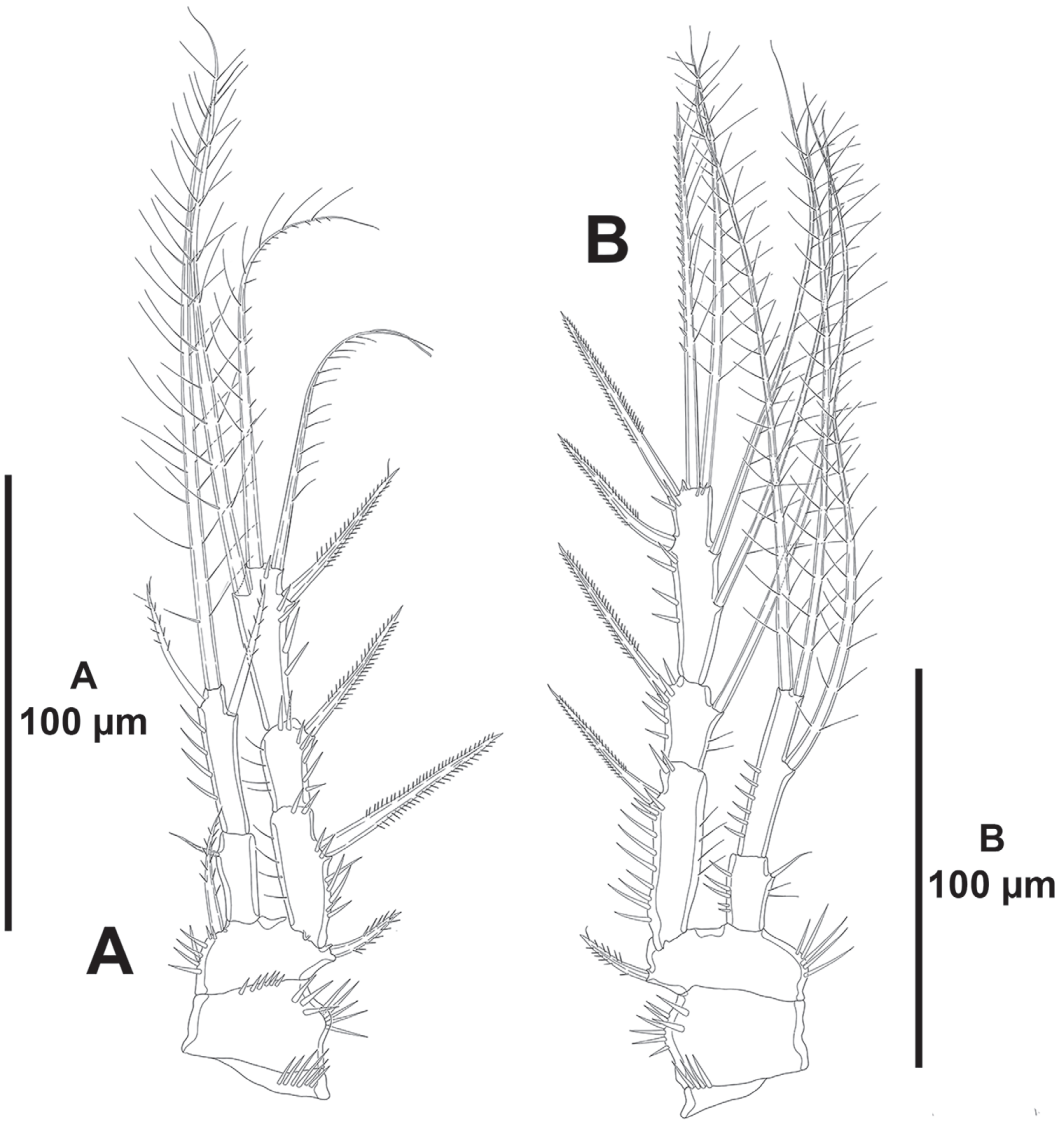
*Mesocletodes unisetosus* sp. n., male paratype. **A** P1, anterior **B** P2, anterior.

*P1* (Fig. 19A). Praecoxa small, with spinular row as shown. Coxa with small median and longer outer spinules. Basis with inner spinules, with outer and inner spines, the former somewhat longer. Exopod tri-segmented; EXP1 as long as EXP3, EXP2 shortest; with spinules as depicted; EXP1 and EXP2 without inner armature; EXP3 with four elements, of which outer and apical element with STE. Endopod bi-segmented, reaching proximal fourth of EXP3; ENP1 seemingly without spinular ornamentation, with one inner seta; ENP2 with few inner spinules, with one outer, one apical and one inner element.

**Figure 20.**
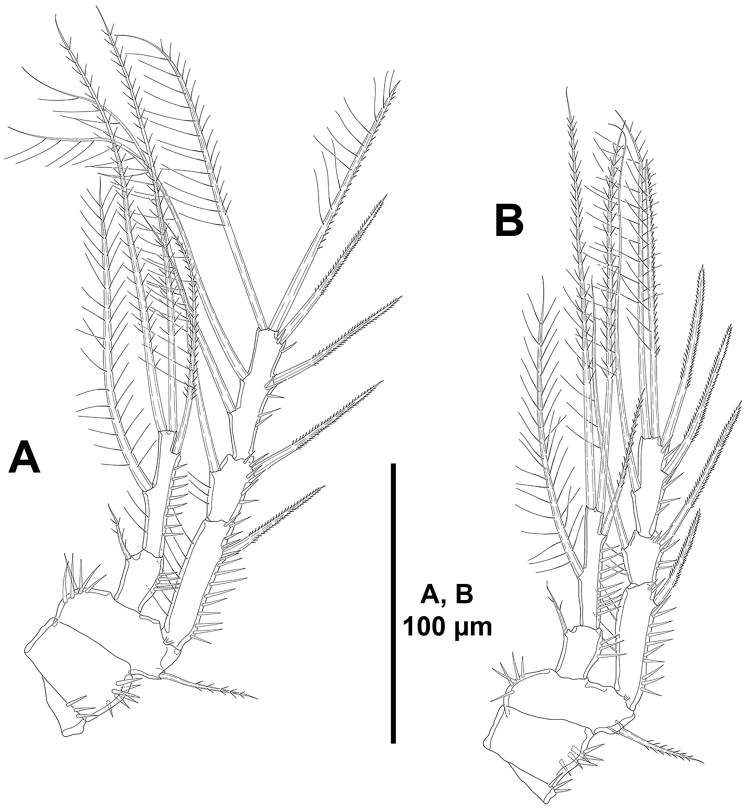
*Mesocletodes unisetosus* sp. n., male paratype. **A** P3, anterior **B** P4, anterior.

*P2–P4* (Figs 19B, 20A, B). Praecoxa small, with few spinules as depicted. Coxa with outer spinules. Basis with inner spinules; outer element of P2 spiniform (Fig. 19B), of P3 and P4 setiform (Fig. 20A, B). Exopod tri-segmented; EXP1 and EXP3 elongated, subequal in length, EXP2 shortest; EXP1 without, EXP2 with inner seta; P2 EXP3 and P3 EXP3 with two outer spines, two apical elements, and two inner setae (Figs 19B, 20A), of P4 EXP3 with two outer spines, two apical elements and one inner seta (Fig. 20B). Endopod of P2 reaching tip of EXP2 (Fig. 19B), of P3 and P4 reaching slightly beyond EXP2 (Fig. 20A, B); ENP1 with one inner small seta (Figs 19B, 20A, B); ENP2 with three (P2; Fig. 19B) and four (P3 and P4; Fig. 20A, B) setae.

**Figure 21.**
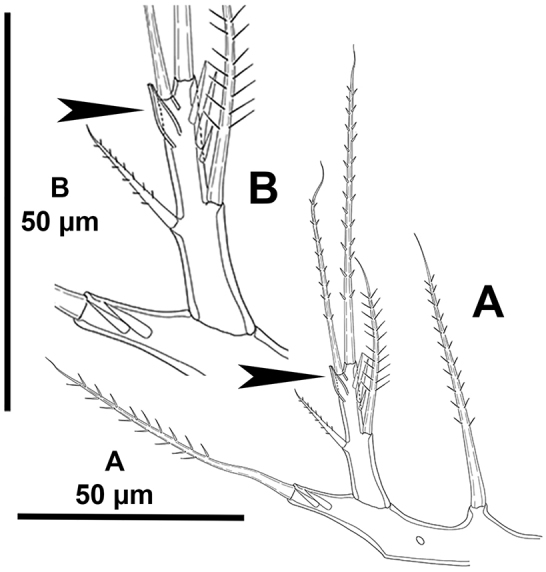
*Mesocletodes unisetosus* sp. n., male paratype. **A** P5, anterior, subdistal tube arrowed **B** exopod of P5 showing distal tube-pore.

*P5* (Fig. 21A, B) with few strong spinules on baseoendopodal setophore. Endopodal lobe poorly developed, with one seta (Fig. 21A). Exopod distinct, long, slender, 3.5 times as long as wide (maximum width measured at its base), with few inner spinules subapically, with two outer, one apical and one inner seta, and one subdistal tube pore.

Armature formula as follows:

**Table:** 

	EXP	ENP
P1	0.0.121	1.111
P2	0.1.222	1.120
P3	0.1.222	1.121
P4	0.1.122	1.121
P5	211+subdistal tube pore	1

###### Description of female.

Unknown.

###### Etymology.

The specific epithet is derived from the Latin prefix *ūni*, meaning one, and the Latin noun *seta*, meaning hair, and refers to the presence of one seta only on the endopodal lobe of the male P5. The name is a noun in the nominative singular.

###### Remarks.

The only species for which the male is known are *M. angolaensis*, *M. elmari* Menzel, 2011, *M. fladensis*, *M. nudus* and *M. unisetosus* sp. n. Of these, the female is known only for *M. elmari*. These species are attributed to Bodin’s (1968) *inermis* group. The males of *M. fladensis*, *M. angolaensis*, and *M. unisetosus* sp. n. possess a dorsal process on the anal somite only and lack mouth parts. *Mesocletodes nudus* and *M. elmari*, lack the dorsal process on the cephalothorax and anal somite, and of these, only the male of *M. nudus* lacks mouth parts. Menzel and George (2009) suggested that the lack of mouth parts in the males of *M. fladensis* and *M. angolaensis*, and consequently in *M. unisetosus* sp. n. and *M. nudus*, might support a monophylum of derived Argestidae (but see below). The same trend has been observed, for example, in the families Aegisthidae (e.g. *Nudivorax* Lee & Huys, 2000, *Scabrantenna* Lee & Huys, 2000, *Andromastax* Conroy-Dalton & Huys, 1999) and Pseudotachidiidae (e.g. Paranannopus Huys, 2009; see Willen 2005). Menzel (2011) suggested that, regardless the developmental stage of the mouth parts (well-developed, strongly reduced or absent), the males of *Mesocletodes* become non-feeding during the last moult as an adaptation to the sparsely populated and oligotrophic deep-sea environments, and could represent a derived character. Also, Menzel (2011) noted that the sexual dimorphism in *M. elmari* (attributed to the *inermis* group), the only species for which both sexes have been described, is expressed, among other characters, in the antennule, P5, P6 and most interestingly, in the armature formula of the P1–P4 ENP2 (but the bi-segmented condition of the rami of the swimming legs is the same in both sexes), and that the mouth parts are present in both sexes regardless of whether the male is non-feeding or not. The males of *M. fladensis*, *M. angolaensis*, and *M. unisetosus* sp. n. are non-feeding and lack mouth parts. Therefore, the option for comparing the armature formula of P1–P4 of the males of these three species only was chosen, viz. with a dorsal process on the anal somite only. Among these species, *M. angolaensis* and *M. unisetosus* sp. n. possess a serrated posterior margin of the cephalothorax, and on the posterior margin of P2-bearing somite to penultimate urosomite, and only *M. fladensis* exhibits posterior spinules instead. An antennary basis is present in *M. angolaensis* and *M. unisetosus* sp. n., and only *M. fladensis* possesses an allobasis. *Mesocletodes angolaensis*, *M. unisetosus* sp. n., and probably *M. fladensis* share the lack of the exopod of the antenna. The Mexican species, *M. unisetosus* sp. n., seems to be more closely related to *M. angolaensis* than to *M. fladensis*. In addition to the characters above shared between these two species, they also share the presence of an inner seta on the P2 and P3 ENP1 (this seta is missing in *M. fladensis*) and the exceedingly elongated caudal rami more than 13 times as long as wide. Briefly, the male of the Mexican species, *M. unisetosus* sp. n., is unique and can be separated from the male of *M. angolaensis*, and *M. fladensis*, by the number of setae on the P5 endopodal lobe (two setae in *M. angolaensis*, and *M. fladensis*, but one seta only in the Mexican *M. unisetosus* sp. n.).

## Discussion

Sars (1909: 290–291) created the genus *Mesocletodes* within the Cletodidae, and presented the diagnosis for the genus based solely on his redescription of the type species, *M. irrasus* (T. Scott & A. Scott, 1894) (Sars 1909: 291–292). Subsequently, Por (1986a) created and diagnosed the family Argestidae to reallocate, among other genera, the genus *Mesocletodes*. For a general historical background on the genus *Mesocletodes* see also Menzel and George (2009) and Vakati et al. (2017).

Sars (1921) described *M. inermis* Sars, 1921, and redescribed *M. monensis* (Thompson, 1893) and *M. abyssicola* (T. Scott & A Scott, 1901), and recognized the relationship between the latter two species (with a dorsal process on the cephalothorax and on the anal somite). He also noted the lack of dorsal processes on the cephalothorax and anal somite of *M. inermis*, but omitted any comment on the relationship of this species and *M. irrasus*. In his key to the species of *Mesocletodes*, Lang (1936) used the presence/absence of a curved dorsal process on the cephalothorax and on the anal somite to separate the species of *Mesocletodes* into two groups, *M. irrasus* and *M. inermis* without dorsal process on the cephalothorax and anal somite *vs. M. brevifurca* Lang, 1936, *M. monensis* and *M. abyssicola* with dorsal process on the cephalothorax and anal somite. Subsequent authors also recognized the relationships amongst those species with a dorsal process on the cephalothorax and on the anal somite (e.g. Soyer 1964, Por 1964a), and amongst those species without such processes (e.g. Por 1964b). It was Bodin (1968) who formally suggested that the species of *Mesocletodes* can be subdivided into two groups, the *abyssicola* group with a dorsal process on the cephalothorax and on the anal somite, and the *inermis* group without a dorsal process on the cephalothorax and anal somite, but noted that such subdivision has no taxonomic value. That Bodin (1968) included in his *abyssicola* group only those species with a dorsal process on the cephalothorax and on the anal somite is evident in his key to the species of *Mesocletodes* where he, for example, included *M. fladensis* Wells, 1965 with a dorsal process on the anal somite only, as part of his *inermis* group. Bodin's (1968) subdivision was accepted and used by subsequent authors (e.g. Coull 1973, Soyer 1975, Schriever 1983, 1985, Bodin 1997).

Wells (1965) described *M. fladensis* from Fladen (in the Scottish sector of the North Sea) based on the male only. This species was described without dorsal process on the cephalothorax, but with a dorsal process on the anal somite, and without mouth parts (i.e. mandibles, maxillules, maxillae and maxillipeds strongly reduced and nontraceable). Later, in his report on five new species of *Mesocletodes* from the North Atlantic Ocean, Schriever (1985) described *M. quadrispinosa* Schriever, 1985, based on four females from the Iceland-Faroe Ridge. *Mesocletodes quadrispinosa* was described with a dorsal process on the cephalothorax, but without a dorsal process on the anal somite. Note that Menzel and George (2009: 252) and Menzel (2011: 47) diagnosed the genus *Mesocletodes* with four setae/spines on P1 EXP3, casting doubts about the relationships and position of *M. quadrispinosa* which was described with three setae only on P1 EXP3.

Por (1986b) presented the description of *M. opoteros* based on five females from the Mozambique Channel between Mozambique and Madagascar. He described *M. opoteros* without a dorsal process on the cephalothorax, but with a dorsal process on the anal somite, and suggested that this species could well belong to a different species-group within *Mesocletodes*, and noted that Bodin’s (1968) division of the genus could change with the discovery of new species. However, upon re-inspection of the type material of *M. opoteros*, Menzel et al. (2011: 862) confirmed the presence of a cuticular dorsal process on the cephalothorax.

Menzel and George (2009) presented the description of *M. angolaensis* from the Angola Basin (Southeastern Atlantic). The only male specimen of this species was described without dorsal process on the cephalothorax, but with a dorsal process on the anal somite, and without mouth parts, similar to what was reported for *M. fladensis* some years earlier. Note that Menzel and George (2009: 253, 254 table 6) erroneously commented on the lack of a dorsal process on the anal somite of *M. fladensis* (see the written description of the species in Wells (1965: 23–24, fig. 77)). They also presented the description of *M. bicornis* Menzel & George, 2009 and *M. dorsiprocessus* Menzel & George, 2009, based on six and two females, respectively, from the Angola Basin (Southeastern Atlantic). They described the females of these two species with a dorsal process on the cephalothorax and anal somite, but also with small dorsal bifid cuticular processes on P3–P5-bearing somites and on the second half of the genital double-somite. In the same paper, Menzel and George (2009) presented the description of *M. meteorensis* Menzel & George, 2009 based on two females from the Angola Basin. This species was described with a dorsal process on the cephalothorax and anal somite, but contrary to the other species of the *abyssicola* group, with the caudal rami barely three times as long as wide. As a result of their investigations, Menzel and George (2009) gave an amended diagnosis of *Mesocletodes* for which they presented four synapomorphies/plesiomorphies, viz. 1) the presence of a strong protrusion with a strong, bipinnate seta pointing backwards on the second antennular segment/without such protrusion and corresponding seta normal, 2) the proximal outer spine of P1 EXP3 reduced/proximal outer spine well developed, 3) the presence of STE’s on the spines of P1 EXP3/without STE, and 4) the mandibular gnathobase with a strong, grinding tooth/gnathobase of normal shape. They did not follow Bodin’s (1968) and Por’s (1986) views regarding the division of the genus, and pooled all the species of *Mesocletodes* with a dorsal cuticular process either on the cephalothorax or on the anal somite, or on both, and with long or short caudal rami, in the *abyssicola* group, arguing that the deviation of Bodin’s (1968) scheme could eventually be regarded as secondary reductions (Menzel and George 2009: 253). Following this reasoning, they suggested the monophyly of the *abyssicola* group as defined by them, for which they proposed three synapomorphies/plesiomorphies: 1) presence of a dorsal cuticular process on the cephalothorax/cephalothorax without dorsal process, 2) presence of a dorsal cuticular process on the anal somite/anal somite without dorsal process, and 3) caudal rami remarkably elongated between seta III and VII/caudal rami not remarkably elongate.

Additionally, Menzel and George (2009) suggested the probable monophyly of, at least, two other groups of species. On one hand, Menzel and George (2009: 254) suggested that the presence of bifid dorsal processes on the P3–P5-bearing somites and on the second half of the genital double-somite (not on the first urosomite as in Menzel and George 2009: 254) of *M. bicornis* and *M. dorsiprocessus* might be of high phylogenetic value to establish a monophylum. On the other hand, Menzel and George (2009: 253–254) suggested that the lack of mouth parts, as observed in *M. fladensis* and *M. angolaensis*, both of the *abyssicola* group *sensu* Menzel and George (2009), can be regarded as derived and therefore, might support a monophylum.

Some years later, Menzel (2011) presented a new and corrected diagnosis for *Mesocletodes* (in their generic diagnosis, Menzel and George (2009) omitted the maxillule, and they described the maxilliped of *Mesocletodes* as stenopodial), presented the sexually dimorphic modifications for the genus with *M. elmari* Menzel, 2011 (without dorsal process on the cephalothorax and anal somite, and with well-developed mouth parts in the male) as model of study, and relegated *M. faroerensis* Schriever, 1985 and *M. thieli* Schriever, 1985 (not *M. thielei* as in Menzel (2011)) as *incertae sedis* within Argestidae due to the presence of an inner seta on P1 EXP2 in the former, and five setae on P1 EXP3 in the latter, and questioned the belonging of *M. arenicola* Noodt, 1952 to this family by the shape and armature of the caudal rami and armature complement of the P1 EXP3. This view is followed here and *M. faroerensis*, *M. thieli* and *M. arenicola* are relegated to *species incertae sedis* within Argestidae. The species presented herein matches the diagnosis of the genus *Mesocletodes* and exhibit the four synapomorphies for the genus.

More recently, Vakati et al. (2017) presented the description of two new species attributable to the *inermis* group, *M. tetrasetosus* Vakati, Thistle & Lee, 2017 and *M. nudus* Vakati, Thistle & Lee, 2017, based on one female and three males, respectively, from the San Diego Trough. Vakati et al. (2017) described the female of *M. tetrasetosus* without dorsal cuticular process on the cephalothorax and anal somite, and with mouth parts. On the other hand, they described the male of *M. nudus* without dorsal processes on the cephalothorax and anal somite, but also, as in the males of *M. fladensis* and *M. angolaensis*, which belong to the *abyssicola* group *sensu* Menzel and George (2009), with atrophied mouth parts. Vakati et al. (2017) presented an amended key to the species of *Mesocletodes* based on Schriever’s (1985) key that, in turn, followed Bodin’s (1968) scheme.

No unequivocal apomorphies have been detected so far to justify the monophyly of the family Argestidae, and its monophyletic status has not yet been demonstrated (George 2004, 2008, 2011). Corgosinho and Martínez Arbizu (2010) suggested that the shape and armature of the maxilla could shed some light on the monophyly of the family. The genus *Mesocletodes* has been diagnosed based on four synapomorphies, but the phylogenetic relationships within the genus are far from clear (Menzel and George 2009). At this point, the *abyssicola* group *sensu* Menzel and George (2009) is considered monophyletic based on the synapomorphic dorsal cuticular process on the cephalothorax and anal somite and the remarkable elongation of the caudal rami between seta III and VII, and the deviations from this scheme have been tentatively attributed to secondary reductions (Menzel and George 2009). The synapomorphic condition of the dorsal process on the cephalothorax and anal somite seems to be well supported since they do not appear in the ground-pattern of Harpacticoida and Argestidae (Menzel and George 2009). Careful inspection of about 800 adult females of *Mesocletodes* with a dorsal process on the cephalothorax, but without an evident dorsal process on the anal somite, revealed the presence of a very small, inconspicuous, dorsal process on the anal somite (Menzel, pers. comm., in litt.). Interestingly, Soyer (1964: 604, fig. D) described the anal operculum of *M. guillei* with a complex ornamentation and with a “crête médiane se terminant par une courte dent….” (Soyer 1964: 604, fig. D). Whether this tooth is homologous to the dorsal process on the anal somite of other species of *Mesocletodes*, remains obscure. Menzel´s observations and the probable unique condition of the dorsal process on the anal somite of *M. guillei*, supports Menzel and George’s (2009) view regarding the definition and monophyly of their *abyssicola* group. Regarding the length:width ratio of the caudal rami, Menzel (2011) hypothesized that the presence of extremely elongated caudal rami in some species of the *inermis* group, viz. *M. elmari*, and in the *abyssicola* group, could be due to convergence. On the other hand, the *inermis* group does not seem to be supported by any synapomorphy.

Given all the above, there seems to be another approach towards the monophyly of the genus *Mesocletodes*. It seems plausible that this genus could eventually be attributed to a new subfamily defined by the four synapomorphies currently known for the genus, viz. the presence of a strong protrusion with a strong, bipinnate seta pointing backwards on the second antennular segment, the proximal outer spine of P1 EXP3 reduced, the presence of STE’s on the spines of P1 EXP3, and the mandibular gnathobase with a strong, grinding tooth, plus the presence of a dorsal cuticular process on the cephalothorax and anal somite.

Under this scheme, the presence of a dorsal process on the cephalothorax and anal somite, and the extreme elongation of the caudal rami between seta III and VII could be regarded as plesiomorphic within the subfamily, and the lack of such processes and the reduction of the caudal rami, as secondary apomorphic reductions. However, this requires more robust, in-depth analyses, accompanied by the diagnosis of this hypothesized subfamily, the re-diagnosis of the genus *Mesocletodes*, and the proposal of a new genus to include all the remaining species. These two genera could be composed as follows:

Hypothetical genus. *M. dorsiprocessus*, *M. bicornis*, and *M. brevisetosus* sp. n.; defined by the synapomorphic bifid dorsal processes on P3–P5-bearing somites and posterior half of genital double-somite. The bifid dorsal process on the cephalothorax could be regarded as autapomorphic for *M. bicornis*. The bifid dorsal process on the anal somite would be regarded as plesiomorphic. The monophyly of this taxon was suggested earlier by Menzel and George (2009).

*Mesocletodes*.– *M. monensis*, *M. abyssicola*, *M. bathybia*, *M. brevifurca*, *M. dolichurus*, *M. katharinae*, *M. meteorensis*, *M. robustus*, *M. soyeri*, *M. opoteros*, *M. simplex* sp. n., *M. quadrispinosa*, *M. fladensis*, *M. angolaensis*, *M. unisetosus* sp. n., *M. irrasus*, *M. inermis*, *M. langi* Smirnov, 1946, *M. makarovi*, *M. glaber* Por, 1964b, *M. guillei*, *M. farauni* Por, 1967, *M. commixtus* Coull, 1973, *M. bodini* Soyer, 1975, *M. carpinei* Soyer, 1975, *M. ameliae* Soyer, 1975, *M. parirrasus* Becker Noodt & Schriever, 1979, *M. sarsi* Becker Noodt & Schriever, 1979, *M. parabodini* Schriever, 1983, *M. trisetosa*, *M. variabilis* Schriever, 1983, *M. kunzi* Schriever, 1985, *M. duosetosus* Schriever, 1985, *M. elmari*, *M. tetrasetosus*, and *M. nudus*; defined by the secondary synapomorphic loss of the dorsal process of the cephalothorax and/or anal somite and reduction of the caudal rami. The elongation of the caudal rami between seta III and VII in some of these species, and the bifid dorsal process on the anal somite of *M. opoteros* are, therefore, plesiomorphic. The presence of the latter in *M. opoteros* and in the previous genus could support a closer relationship between these two taxa. Four species groups without taxonomic value, for which no apomorphies have been detected, can be envisaged based on the presence/absence of a dorsal cuticular process on the cephalothorax and/or anal somite:

I *M. abyssicola*, *M. brevifurca*, *M. bathybia*, *M. dolichurus*, *M. katharinae*, *M. meteorensis*, *M. monensis*, *M. opoteros*, *M. robustus*, *M. simplex* sp. n., and *M. soyeri*

II *M. quadrispinosa*

III *M. angolaensis*, *M. fladensis*, and *M. unisetosus* sp. n.

IV *M. ameliae*, *M. bodini*, *M. carpinei*, *M. commixtus*, *M. duosetosus*, *M. elmari*, *M. farauni*, *M. glaber*, *M. guillei*, *M. inermis*, *M. irrasus*, *M. kunzi*, *M. langi*, *M. makarovi*, *M. nudus*, *M. parabodini*, *M. parirrasus*, *M. sarsi*, *M. tetrasetosus*, *M. trisetosa*, and *M. variabilis*.

Group I includes Bodin’s (1968) *abyssicola* group. Group II contains only *M. quadrispinosa*, but as noted above, the position of this species is doubtful. Group III contains those species with a dorsal cuticular process on the anal somite only. Group IV contains Bodin’s (1968) *inermis* group. No synapomorphies have been detected so far for each of these groups. On the other hand, Menzel and George (2009) suggested that the lack of mouth parts in *M. fladensis* and *M. angolaensis*, but also in *M. unisetosus* sp. n. and *M. nudus* could support a monophylum of derived Argestidae. However, if the monophyly of the above groups is confirmed, the lack of mouth parts in group III and group IV could be attributed to convergence.

Finally, it is worth mentioning the presence of some other undescribed forms related to *M. angolaensis*, *M. bicornis*, *M. brevisetosus* sp. n., *M. dorsiprocessus*, *M. meteorensis*, *M. simplex* sp. n., and *M. unisetosus* sp. n. in the Clarion-Clipperton Fracture Zone in the Pacific Ocean (Samantha Tong Jia Wen, Tropical Marine Science Institute, National University of Singapore, pers. comm.).

## Supplementary Material

XML Treatment for Mesocletodes brevisetosus

XML Treatment for Mesocletodes simplex

XML Treatment for Mesocletodes unisetosus
